# Rarefaction and extrapolation of species richness using an area‐based Fisher's logseries

**DOI:** 10.1002/ece3.3509

**Published:** 2017-10-23

**Authors:** Youhua Chen, Tsung‐Jen Shen

**Affiliations:** ^1^ Department of Renewable Resources University of Alberta Edmonton AB Canada; ^2^ Chengdu Institute of Biology Chinese Academy of Sciences Chengdu China; ^3^ Institute of Statistics & Department of Applied Mathematics National Chung Hsing University Taichung Taiwan

**Keywords:** area dependency, biodiversity comparison, richness estimation, sampling effect, statistical ecology

## Abstract

Fisher's logseries is widely used to characterize species abundance pattern, and some previous studies used it to predict species richness. However, this model, derived from the negative binomial model, degenerates at the zero‐abundance point (i.e., its probability mass fully concentrates at zero abundance, leading to an odd situation that no species can occur in the studied sample). Moreover, it is not directly related to the sampling area size. In this sense, the original Fisher's alpha (correspondingly, species richness) is incomparable among ecological communities with varying area sizes. To overcome these limitations, we developed a novel area‐based logseries model that can account for the compounding effect of the sampling area. The new model can be used to conduct area‐based rarefaction and extrapolation of species richness, with the advantage of accurately predicting species richness in a large region that has an area size being hundreds or thousands of times larger than that of a locally observed sample, provided that data follow the proposed model. The power of our proposed model has been validated by extensive numerical simulations and empirically tested through tree species richness extrapolation and interpolation in Brazilian Atlantic forests. Our parametric model is data parsimonious as it is still applicable when only the information on species number, community size, or the numbers of singleton and doubleton species in the local sample is available. Notably, in comparison with the original Fisher's method, our area‐based model can provide asymptotically unbiased variance estimation (therefore correct 95% confidence interval) for species richness. In conclusion, the proposed area‐based Fisher's logseries model can be of broad applications with clear and proper statistical background. Particularly, it is very suitable for being applied to hyperdiverse ecological assemblages in which nonparametric richness estimators were found to greatly underestimate species richness.

## 1 INTRODUCTION

Fisher's logseries and its key element, Fisher's alpha index (Fisher *et al*. 1943), originally developed by the founder of biostatistics R.A. Fisher, are widely used in ecological studies (Kempton & Taylor 1974; Rice & Demarais 1996). In particular, they have been broadly applied to estimate species richness (Harte *et al*. 2008; Harte & Kitzes 2015; Slik *et al*. 2015; ter Steege *et al*. 2017) and accordingly the extinction of species (Gilbert *et al*. 2006; Halley & Iwasa 2011; Kitzes & Harte 2015). However, the application on richness estimation has generated some controversy (Chao & Chiu 2016), partially because many parametric models could fit the same empirical data equally well (McGill 2003, 2006), and partially because of the overestimation risk of species richness using parametric estimators (Xu *et al*. 2012; ter Steege *et al*. 2017). Therefore, nonparametric richness estimators (Chao 1984; Colwell *et al*. 2012; Chao & Chiu 2016; Hsieh *et al*. 2016), instead, have gained much more attention in empirical applications.

However, the biggest challenge confronted by most nonparametric richness estimators is that they can only provide lower bounds of species richness (Chao & Lin 2012; Chiu *et al*. 2014), greatly underestimating regional species richness (Chao *et al*. 2016). For example, two recent empirical studies (Slik *et al*. 2015; ter Steege *et al*. 2017) showed that no popular nonparametric estimators could predict a reasonable number of tropical tree species, as all of them predicted richness values that were too small and largely deviated from ecologists’ estimation. Other similar works (Chiarucci *et al*. 2003; Xu *et al*. 2012) reached a similar conclusion that nonparametric methods are not suitable to estimate species richness in highly diverse ecological communities.

A thorough investigation of relevant statistical properties of a parametric method, like the Fisher's alpha index, is necessary when ecologists want to correctly apply it in the empirical setting. However, the derivation of Fisher's alpha index from the negative binomial model (NBD) (Fisher *et al*. 1943) is statistically formidable, and consequently, it is not a standard probability mass function. Further, application of the ordinary NBD in developing the alpha index in Fisher's original work implies that this index does not explicitly take into account the compounding effect of sampling areas, although the sampling area size is indirectly related to the community size. In such a context, it is inappropriate to directly compare species richness predicted by the alpha diversity index between different ecological assemblages sampled from areas of varying sizes, as larger areas would always tend to have higher species diversity (Hurlbert 1971; Gotelli & Colwell 2001; Hubbell 2015; Slik *et al*. 2015). Last but not least, previous empirical studies (Schulte *et al*. 2005; Slik *et al*. 2015; ter Steege *et al*. 2017) which applied Fisher's logseries to estimate species richness did not provide 95% confidence interval for the estimated richness. One possibility for this is because the variance formula provided in Fisher's original paper (Fisher *et al*. 1943) is biased and will result in very small variance (and accordingly very unreasonably narrow 95% confidence interval) for the estimated regional species richness, which will be demonstrated in detail later.

To overcome the abovementioned problems and derive a standard probability function for Fisher's logseries when applied to ecological research, we used a truncated NBD (TNBD) to deduce the logseries distribution. This new logseries model is a standard probability mass function, explicitly incorporating the area effect of the sampling site and thus satisfying the fact that Fisher's alpha index changes when the sampling area varies (Hubbell 2015; Slik *et al*. 2015). Given these virtues of the new model, the standard rarefaction and extrapolation processes can be conducted. More importantly, in comparison with the original Fisher's logseries, our area‐based model can offer an asymptotically unbiased estimation of the variance and accordingly the correct 95% confidence interval of the estimated species richness (for either extrapolation or rarefaction).

In summary, the central goals of this study were to address the following questions: When one has species abundance distribution (SAD) data from local sampling sites with varying area sizes and one also confirms that they are very likely to follow Fisher's logseries, what would the expected regional species richness be for a given larger area under Fisher's distribution assumption? What are the 95% confidence intervals (CIs) when conducting rarefaction or extrapolation of species richness using area‐dependent Fisher's alpha? How can ecologists determine when Fisher's logseries could be applied?

## 2 MATERIALS AND METHODS

### 2.1 A review of the original Fisher's logseries model

Following Fisher *et al*. (1943)'s annotations, suppose there are *S* species in a community where each species has an abundance, *N,* following an NBD with the probability mass function (pmf) as

(1) $$P\left(N=n\right)=\frac{\Gamma (k+n)}{\Gamma (k)\Gamma (n+1)}\frac{p^{n}}{{\left(1+p\right)}^{k+n}},\;\;\;n=0,1,2\ldots ;$$

where *p* > 0 and *k* > 0 are two parameters, and the latter one is commonly called an aggregation parameter. Fisher *et al*. (1943) took the limit of *k* → 0 of this NBD model (Equation (1)) to derive his logseries model. However, there is a problem: when *k* → 0, *P*(*N* = 0) → 1 (because Γ(*k*) cancels out in the denominator and numerator in the first term on the right side in Equation (1)) while *P*(*N* = *n*) → 0 (because Γ(k)/Γ(k+n)→0) for any *n* ≥ 1; see the Supporting Information in detail. This means that the probability mass is degenerated or fully concentrated at the zero point as *k* → 0. In other words, the limit for *k* → 0 in Equation (1) makes it impossible for a species to occur in the studied sample (i.e., it is unseen in the sample). Consequently, Fisher *et al*. (1943, p. 54) remarked “The limiting value *k* = 0 cannot occur in cases where the frequency at zero is observable, for the distribution would then consist wholly of such cases” and thus discarded such a way to derive the logseries model.

To avoid this unseen species problem and as ecologists are only concerned with species that can be seen or observed in a studied sample, Fisher *et al*. (1943) let 1/Γ(k) be a finite constant α when *k* → 0, then Fisher *et al*. (1943) proposed that, as *k* → 0 and by ignoring the zero abundance case, a logseries distribution has a form as follows:

(2) $$\alpha \frac{x^{n}}{n},\;n=1,2,\;...;$$

where x=p/(1+p), and the parameter α was named “alpha” diversity. These parameters can, respectively, be estimated using the equalities *S*_0_ = − α ln (1 − *x*) and $M_{0}=\alpha /\left(1-x\right)$ (Fisher *et al*. 1943). Here, *S*_0_ represents the number of species and *M*_0_ the total number of individuals observed in the studied sample.

### 2.2 The proposed area‐based Fisher's logseries model

Suppose one has a finite studied region, and its area size is denoted by *A*; then, a TNBD instead of the ordinary NBD in Equation (1) is employed to account for all species necessarily being present in the targeted region *A,* and its pmf is as follows:

(3) $$P\left(N_{A}=n|A,\;\;k,\;\;\omega \right)=C\frac{\Gamma (k+n)}{\Gamma (k)\Gamma (n+1)}{\left(\frac{\omega }{\omega +A}\right)}^{k}{\left(\frac{A}{\omega +A}\right)}^{n},\;n=1,2\ldots ;$$

where $C={\left(1-{\left(\frac{\omega }{\omega +A}\right)}^{k}\right)}^{-1}$. Note that the pmf (Equation (2)) can be derived from a gamma‐Poisson mixture model and specifically be calculated from

$$P\left(N_{A}=n|A,\;\;k,\;\;\omega \right)=\frac{\int_{0}^{\infty }\frac{{\left(A\lambda \right)}^{n}e^{-A\lambda }}{n!}\frac{\omega^{k}\lambda^{k-1}e^{-\lambda \omega }}{\Gamma (k)}d\lambda }{1-\int_{0}^{\infty }e^{-A\lambda }\frac{\omega^{k}\lambda^{k-1}e^{-\lambda \omega }}{\Gamma (k)}d\lambda },\;n=1,2\ldots$$

By excluding the zero abundance of species in the studied region, this truncated model avoids the odd situation at *n* = 0 when *k* → 0 encountered in the NBD used by Fisher *et al*. (1943). Using this model with *k* → 0, the limiting distribution can be derived by:

(4) $$\begin{array}{cc} \phi (n|A,\;\;\omega )= & \lim_{k\to 0}P\left(N_{A}=n|A,\;\;k,\;\;\omega \right)={\left[\ln\left(1+\frac{A}{\omega }\right)\right]}^{-1}\left(\frac{1}{n}\right){\left(\frac{A}{\omega +A}\right)}^{n},\\ & n=1,2,\;...; \end{array}$$

where $x_{A}=A/\left(\omega +A\right)$ and $\alpha_{A}={\left[\ln\left(1+A/\omega \right)\right]}^{-1}$ are defined so as to correspond to *x* and α in the original Fisher's logseries distribution (Equation (1)). Obviously, one can see that the regional area, *A,* can be part of both parameters (*x*_*A*_ and α_*A*_); this is the reason that we call our model an area‐based Fisher's model. Note that Engen (1978) also gave a zero‐truncated logseries distribution that differs from the variant in Equation (2) taking the area size *A* into consideration.

Because Equation (2) is a standard probability mass function (i.e., $\sum_{n=1}^{\infty }\phi (n|A,\;\;\omega )=1$), α_*A*_ in our study is in a range of 0–1 and thus differs from the original Fisher's alpha (which is directly related to species richness). Therefore, we renamed our α_*A*_ as the normalized Fisher's alpha index. As *x*_*A*_ is a function of α_*A*_, our model can be further simplified to contain only one unknown parameter, α_*A*_, resulting in the following form:

(5) $$\phi \left(n|\alpha_{A}\right)=\frac{\alpha_{A}}{n}{\left(1-e^{-1/\alpha_{A}}\right)}^{n};$$

the mean and variance of which are, respectively, given by $\alpha_{A}\left(e^{1/\alpha_{A}}-1\right)$ and $\alpha_{A}\left(e^{1/\alpha_{A}}-1\right)\left[\left(1-\alpha_{A}\right)e^{1/\alpha_{A}}+\alpha_{A}\right]$ We can extend our model to a local sampling area that is a part of region *A*, as conducting a comprehensive census over the entire region *A* is unrealistic. In comparison, surveying a local area with a size *a* from region *A* is practical and less labor‐intensive in the field. To do this, we defined the number of individuals of each species observed in a local sample of area *a* as *N*_*a*_; then, the probability function *N*_*a*_ can be derived from the TNBD in Equation (2) as

(6) $$P\left(N_{a}=n|a,\;\;A,\;\;k,\;\;\omega \right)=\left\{\begin{array}{c} C\left[{\left(\frac{\omega }{\omega +a}\right)}^{k}-{\left(\frac{\omega }{\omega +A}\right)}^{k}\right],\;n=0\\ C\frac{\Gamma (k+n)}{\Gamma (k)\Gamma (n+1)}{\left(\frac{\omega }{\omega +a}\right)}^{k}{\left(\frac{a}{\omega +a}\right)}^{n},\;n=1,2,\ldots \end{array}\right.$$

As a result, the limiting distribution of *N*_*a*_ as *k* → 0 can be derived from Equation (5), and its probability function is as follows:

(7) $$\phi \left(n|\alpha_{a},\alpha_{A}\right)=\lim_{k\to 0}P\left(N_{a}=n|a,\;\;A,\;\;k,\;\;\omega \right)=\left\{\begin{array}{c} (1-\alpha_{A}/\alpha_{a}),\;n=0\\ \frac{\alpha_{A}}{n}(1-e^{-1/\alpha_{a}}{)}^{n},\;n=1,2,\;... \end{array}\right.$$

Detailed derivation of the above limiting distribution when *k* → 0 is provided in the Supporting Information. Note that the probability function in Equation (6) can theoretically converge to the probability function in (5) as $\phi \left(0\left|\alpha_{A},\alpha_{a}\right.\right)=0$ when *a* = *A*, which is equivalent to conducting a census over the entire studied region. This convergent behavior is also in response to why we employed a TNBD in Equation (2), as the unseen probability of a species in the studied region (e.g., the entire surface of the Earth) has to vanish if it can be comprehensively censused (ignoring time‐consuming speciation events during the census period).

### 2.3 Parameter estimation

Let *f*_*k*_ be the number of species with *k* individuals observed in the sample and *f*_0_ be the number of species unseen in sample *a* but present in the studied region *A*. Note that only *f*_*k*_, *k* ≥ 1 (frequency counts) can be observed in the sample. As a result, the likelihood function, based on frequency counts, is given by

(8) $$\begin{array}{cc} & L(S_{A},\;\;\omega |f_{1},\;...,\;f_{\tau })\\ & =\frac{\Gamma \left(S_{A}+1\right)}{\Gamma \left(S_{A}-S_{a}+1\right)\prod_{j=1}^{\tau }\Gamma \left(f_{j}+1\right)}{\left[\phi (0\left|\alpha_{a},\alpha_{A}\right.)\right]}^{S_{A}-S_{a}}\prod_{n=1}^{\tau }{\left[\phi (n\left|\alpha_{a},\alpha_{A}\right.)\right]}^{f_{n}}\\ & =\frac{\Gamma \left(S_{A}+1\right)}{\Gamma \left(S_{A}-S_{a}+1\right)\prod_{j=1}^{\tau }\Gamma \left(f_{j}+1\right)}{\left[\frac{\alpha_{A}}{\alpha_{a}}\right]}^{S_{a}}{\left[1-\frac{\alpha_{A}}{\alpha_{a}}\right]}^{S_{A}-S_{a}}\left(\prod_{n=1}^{\tau }{\left[\frac{1}{n}\right]}^{f_{n}}\right)\alpha_{a}^{S_{a}}{\left(1-e^{-1/\;\alpha_{a}}\right)}^{M_{a}} \end{array}$$

where $M_{a}=\sum_{n=1}^{\tau }nf_{n}$ and τ = max{*k*:*f*_*k*_, *k* ≥ 1}. *S*_*a*_ and *M*_*a*_, respectively, represent the number of species and number of total individuals observed in local area *a*, containing all information for estimating unknown parameters. They are the so‐called sufficient statistics by Ronald A. Fisher. As a result, the maximum‐likelihood estimates (MLEs) of *S*_*A*_ and ω, by maximizing the likelihood function in Equation (1), can be equivalently solved from the following equations:

(9) $$\left\{\begin{array}{c} \frac{M_{a}}{S_{A}}=E\left(N_{a}\right)=\alpha_{A}\left(e^{1/\;\alpha_{a}}-1\right)\\ S_{a}=E\left(S_{a}\right)=S_{A}\left(\alpha_{A}/\;\alpha_{a}\right) \end{array}\right.$$

These MLE‐derived equalities in Equation (1) can also be deduced when applying the moment of methods to Equation (6). Variances of ${\hat{S}}_{A}$ and $\hat{\omega }$ can accordingly be estimated from diagonal elements of the inverse of the observed information matrix. Then, the normalized diversity index, α_*A*_, for the entire region can be estimated by

(10) $${\hat{\alpha }}_{A}=1/\;\ln(1+A/\hat{\omega })$$

### 2.4 Interpolation and extrapolation of species richness

Consider an interpolated or extrapolated area of size A*. Interpolation (0 < A* ≤ *a*) or extrapolation (*A* ≥ A* ≥ *a*) of species richness from local area *a* can be estimated by the following estimator:

(11) $${\hat{S}}_{A^{*}}=S_{a}{\hat{\alpha }}_{a}/{\hat{\alpha }}_{A^{*}}.$$

Applying the variance decomposition formula to $Var\left({\hat{S}}_{A^{*}}\right)$, which is conditional on *S*_*a*_, we can estimate the variance as

(12) $$V\hat{a}r\left({\hat{S}}_{A^{*}}\right)=S_{A^{*}}^{2}{\left(\frac{{\hat{\alpha }}_{a}a}{{\hat{\omega }}^{2}+{\hat{\omega }}^{2}a}-\frac{{\hat{\alpha }}_{A^{*}}A^{*}}{{\hat{\omega }}^{2}+{\hat{\omega }}^{2}A^{*}}\right)}^{2}V\hat{a}r\left(\hat{\omega }\right)+{\left(\frac{{\hat{S}}_{A^{*}}}{S_{a}}\right)}^{2}V\hat{a}r\left(S_{a}\right),$$

where $V\hat{a}r\left(S_{a}\right)=S_{a}\left(1-S_{a}/{\hat{S}}_{A}\right)$.

The variance $V\hat{a}r\left({\hat{S}}_{A^{*}}\right)$ estimated using Equation (3) for our proposed area‐based model differs from the original one proposed by Fisher *et al*. (1943), the calculation details of which are presented in the additional method section of the Supporting Information.

### 2.5 Numerical tests

As Fisher's logseries distribution in Equation (1) is not a standard probability distribution and lacks a specific sampling framework, it is difficult to conduct numerical tests with the model and estimate related parameters. In contrast, our area‐based model (Equation (6)) has a standard parametric probability distribution with an explicit sampling structure (i.e., local versus regional models). Accordingly, the asymptotic properties of parameter estimation in our model are clear.

Therefore, we conducted extensive simulations to demonstrate the performance of regional richness estimation (i.e., extrapolation) and checked what we found using the proposed area‐based model. First, we simulated sampling data from the proposed area‐based Fisher's logseries in Equation (6). Details of the simulation algorithm are presented in additional methods section of the Supporting Information. In our simulation, the regional species richness, the parameter ω, and the regional area size information can vary and were given when simulating species abundances in local sampling area *a* (the size of which was fixed as 1 in all scenarios).

The regional species richness is allowed to vary as *S*_*A*_ = 500, 2,000, or 6,000. The area size of the region can vary as *A *=* *100, 1,000, 10,000, or 50,000. Finally, we let parameter ω vary as ω = 0.005, 0.01, or 0.05. Based on this, we had 3 × 4×3 = 36 configurations when simulating local species diversity data for subsequent analyses and comparisons.

In addition to simulating data following the proposed model, we further simulated data for another two models of species abundance distribution. One is from the pmf in Equation (6), that is, TNBD, with letting *k* be 1, 0.5, 0.1, and 0.01 and fixing ω =0.01 and *A *=* *100; note that this model will approach to the area‐based logseries model when *k* becomes small. As a special case, TNBD is the same as the geometric series model when *k *=* *1.

The other model considered simulating species abundance data approximately following a lognormal distribution. To take the area effect into consideration while to ensure that all species have positive probabilities to exist in the study region, given the intensity λ that is related to the mean abundance of a species, we let *N*_*A*_ follow a zero‐truncated Poisson distribution having the conditional pmf as follows:

$$P\left(N_{A}=n|\lambda ,\;\;A\right)=\frac{{\left(A\lambda \right)}^{n}e^{-A\lambda }}{n!\left(1-e^{-A\lambda }\right)},n\geq 1.$$

A sample with area *a* taken from the entire region, the abundance of a species in the sample, *N*_*a*_, can be derived from the pmf of *N*_*A*_ and has the condition pmf as

$$P\left(N_{a}=n|\lambda ,\;\;a,\;\;A\right)=\left\{\begin{array}{c} \frac{e^{-a\lambda }\;\;-\;\;e^{-A\lambda }}{1-e^{-A\lambda }},\;\;n=0\\ \frac{\left(a\lambda \right){}^{n}e^{-a\lambda }}{n!(1-e^{-A\lambda })},\;\;n\geq 1 \end{array}.\right.$$

We then considered that λ follows a lognormal distribution transformed from a normal distribution with mean μ and standard deviation σ, where μ was fixed at zero and σ varied from 1.5 to 3 with an increment 0.5 in the simulation study. For simplicity, TPLN (μ*,* σ) is used to signify this model for truncated Poisson‐lognormal distribution.

For each configuration or combination, we independently simulated 5,000 local species diversity data (abundance and number) and then measured relevant quantities, including an average of the estimated species richness and the sample standard error (*SE*) computed by the simulated data. Additionally, the averaged estimated *SE* of species richness was computed using over 5,000 simulated data for each estimator so the performance of the estimators could be compared. A reasonable variance estimator was determined whether its estimated *SE* was very close to the sample *SE*.

For each of the above generated local species abundance data, we fit our proposed area‐based Fisher's alpha model to the local data and the fitted model was used to perform regional species richness estimation (extrapolation). To demonstrate the predictive power of the proposed parametric model, regional species richness was also estimated by applying three commonly used nonparametric methods to the simulated local data for comparison, including the Chao1 estimator (${\hat{S}}_{Chao1}$) (Chao 1984), abundance‐based coverage estimator (ACE: ${\hat{S}}_{\mathrm{ACE}}$) (Chao & Lee 1992), and first‐order jackknife estimator (${\hat{S}}_{Jk1}$) (Burnham & Overton 1978; Heltshe & Forrester 1983). Their methods of calculating richness extrapolation and the corresponding variance formulas are provided in the Supporting Information.

Comparing species diversities between different local communities is very common in ecology. However, samples from different local communities often differ in their sampling areas (and individual sample numbers as well). Therefore, richness interpolation or rarefaction should be performed when ecologists want to compare and rank the species diversity status of different samples (Hurlbert 1971; Heck *et al*. 1975; Soetaert & Heip 1990; Gotelli & Colwell 2001). In our study, the proposed index α_*A*_ takes both the local sampling area and the entire region into account; thus, it is like a species–area relationship (Gleason 1922). However, contrary to conventional species–area relationships, our area‐based model only requires observed individual and species numbers as inputs to establish the relationship between sampling area and species richness.

Here, apart from the richness extrapolation, we again also performed local species richness rarefaction using the proposed area‐based model, through numerical simulation. The purpose of performing area‐based rarefaction was to compare and rank local species diversity statuses for three theoretical sites (L1, L2, and L3) from a region with a total area size *A *=* *30. Suppose that we had conducted field surveys of these three local sites, and the following data on the local species richness, community size, and sampling area size had been gathered: site L1 had species number = 100, total individual number = 5,000, and sampling area size = 15; site L2 had species number = 50, total individual number = 2,000, and sampling area size = 1; and site L3 had species number = 80, total individual number = 2,000, and sampling area size = 2. We also assumed that we knew that the local SADs in these three sites followed a Fisher's logseries distribution. At first glance, it seems that L1 has the highest species richness, followed by L3 and L2. Moreover, because both L2 and L3 had the same total number of individuals and the species richness–area ratio is higher for L2, it seems that L2 might have higher diversity than site L3.

However, as previously outlined, we cannot directly compare species richness levels of these sites because their sampling area sizes (and also sampling individual numbers) differ. Therefore, we fit our area‐based Fisher's alpha model into these three local samples and then performed species richness rarefaction so as to rank species diversity among the three hypothetical sites at a given baseline area (e.g., area = 10). Through our area‐based rarefaction, we can show that it was not true that site L1 had the highest species richness. Also, we can show that the species richness at site L2 was not higher than that at site L3 as expected earlier.

### 2.6 An empirical test

We fit our proposed area‐based Fisher's alpha model to tree species in interior (species number = 371, total individual number = 2,174) and edge (species number = 332, total individual number = 1,966) areas, respectively, from 12 fragments of Brazilian Atlantic forests (Magnago *et al*. 2014). The original species frequency count data from their paper are summarized in Table 1. In their original data, for each of the 12 fragments, an edge transect and an interior transect were sampled. Each transect was composed of ten 10 × 10‐m plots. Species richness was extrapolated to estimate species richness at a broader spatial scale (combining all sampling plots from both edge and interior areas = 2.4 ha) and the entire region (the 12 fragments, which had a size of *A *=* *67,282.16 ha). As a comparison, two nonparametric methods used above, including Chao1 and ACE estimators, were also performed and compared.

**Table 1:** Original species abundance distribution data in terms of species frequency counts reported by Magnago *et al*. (2014) for the interior (1.2 ha), edge (1.2 ha), and combined areas (2.4 ha) in 12 fragments of Brazilian Atlantic forests

Habitat	*f*_1_	*f*_2_	*f*_3_	*f*_4_	*f*_5_	*f*_6_	*f*_7_
Edge	115	49	38	28	14	11	13
Interior	128	49	42	33	19	17	7
Edge+Interior	115	57	32	41	26	23	15

Habitat	*f*_8_	*f*_9_	*f*_10_	*f*_11_	*f*_12_	*f*_13_	*f*_14_
Edge	5	6	6	3	4	3	5
Interior	9	7	7	6	3	3	3
Edge+Interior	15	13	10	4	4	6	6

Habitat	*f*_15_	*f*_16_	*f*_17_	*f*_18_	*f*_19_	*f*_20_	*f*_21_
Edge	2	5	2	2	2	2	1
Interior	4	4	2	2	3	4	6
Edge+Interior	5	6	4	2	6	2	1

Habitat	*f*_22_	*f*_23_	*f*_24_	*f*_25_	*f*_26_	*f*_27_	*f*_28_
Edge	0	2	0	1	0	1	1
Interior	0	2	0	1	0	2	1
Edge+Interior	2	2	4	2	3	3	1

Habitat	*f*_29_	*f*_30_	*f*_31_	*f*_32_	*f*_33_	*f*_34_	*f*_35_
Edge	0	1	0	1	0	0	0
Interior	0	1	0	1	0	1	1
Edge+Interior	1	1	1	2	1	0	0

Habitat	*f*_36_	*f*_37_	*f*_38_	*f*_39_	*f*_40_	*f*_41_	*f*_42_
Edge	2	1	0	0	0	1	0
Interior	0	0	0	0	0	0	0
Edge+Interior	3	1	1	3	1	1	2

Habitat	*f*_43_	*f*_45_	*f*_46_	*f*_49_	*f*_51_	*f*_52_	*f*_53_
Edge	0	1	1	1	0	0	0
Interior	0	0	0	0	0	1	0
Edge+Interior	1	0	2	0	2	1	2

Habitat	*f*_55_	*f*_68_	*f*_79_	*f*_89_	*f*_104_	*f*_110_	*f*_121_
Edge	0	0	0	1	0	1	0
Interior	0	0	0	0	0	0	0
Edge+Interior	1	1	1	0	1	0	1

Habitat	*f*_123_	*f*_140_	*f*_159_	*f*_181_	*M*_*a*_	*S*_*a*_	
Edge	0	0	0	0	1,966	332	
Interior	1	1	0	0	2,174	371	
Edge+Interior	0	0	1	1	4,140	443	

Moreover, as we have observed data combined from the interior and edge areas (i.e., at the augmented 2.4‐ha spatial scale, total species number = 443, total individual number = 4,140) (Magnago *et al*. 2014), the rarefaction of species richness for the 1.2‐ha interior or edge areas from the combined area (i.e., the augmented 2.4‐ha area) can be performed and validated as well. In contrast to the richness extrapolation using Chao1 and ACE, Hurlbert (1971)'s individual‐based and Coleman (1981)'s area‐based methods were correspondingly applied for comparison.

To conduct goodness‐of‐fit tests of our proposed model when applied to Magnago et al.'s empirical dataset (for either edge, interior, or the augmented combined areas), we utilized both the Kolmogorov–Smirnov (KS) and chi‐squared (χ^2^) tests (Arnold & Emerson 2011). In particular, the KS test has to be adjusted because species abundance is a discrete variable (Arnold & Emerson 2011).

Other than verifying the predictive power of the species richness interpolation associated with the 95% CIs using the fitted area‐based models to cover the true observed species richness in the 1.2‐ha edge and interior areas (and extrapolation to the 2.4‐ha augmented areas), we extrapolated the species richness using the fitted area‐based models up to the entire region (i.e., the sum of all 12 fragments, with an area size 67,282.16 ha), even though the true species richness at this large spatial scale was unknown. Because we were estimating regional species richness here, all three nonparametric methods, including Chao1, ACE, and first‐order jackknife estimators, were applicable and implemented for comparison.

## 3 RESULTS

**Figure 1:**
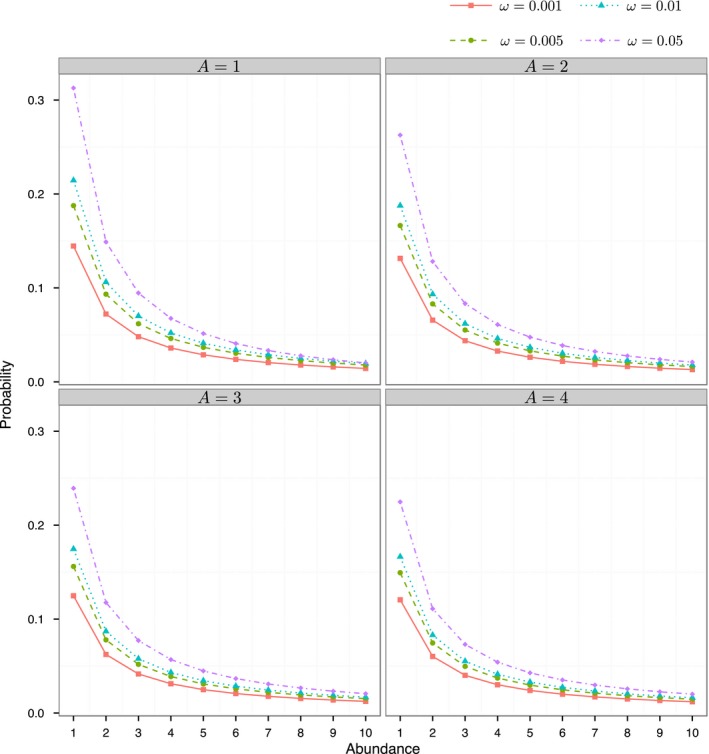
Curve shape patterns of the proposed area‐based Fisher's logseries model as a function of ω and regional area *A*

The curved shape of Fisher's logseries predicts more rare species if parameter ω is larger or the regional area size is smaller (Figure 1). Such patterns can theoretically be interpreted by Equation (6). No matter what values of ω and regional area *A* are used in Figure 1, the ratio of the relative abundances for *n *=* *1 and *n *=* *2 was close to two, which is a key feature predicted by Fisher's logseries model. Original data (Table 1) on the species frequency counts reported by Magnago *et al*. (2014) empirically showed that this ratio could exist in field surveys.

When data were simulated from the area‐based logseries model, numerical simulation results showed that the nonparametric richness estimators (Chao1, ACE, and first‐order jackknife) always underestimated the true regional species richness to large extents (Table 2 and Tables S1–S3). Regardless of the true values of regional species richness, regional area size, and parameter ω given in the simulations, the estimated regional species richness using the proposed area‐based method was consistently and statistically close to true values (Table 2 and Tables S1–S3). Furthermore, compared to Fisher's original logseries model and other estimators, for each simulation configuration, the coverage percentage (CP) of the 5,000 simulation in which the 95% CIs covered the “true” species richness in the region for the proposed area‐based model was always closest to the nominal value of 0.95 (Table 2 and Tables S1–S3).

**Table 2:** Comparisons of the performance of estimating regional species richness of different statistical methods based on the available species diversity information from simulated local samples. The true value indicates that the predesigned species assemblage in regional area *A* had a richness *S,* and the abundance of all species followed a logseries distribution with the given parameter, ω, for the simulation. *S*_*a*_ is the species richness observed in local sample *a* (area size = 1) averaged from 5,000 simulations. Regional species richness was estimated by four methods, including three nonparametric methods and our proposed area‐based method (${\hat{S}}_{A}$). CP is the coverage percentage of the 5,000 generated datasets in which the 95% confidence intervals covered the “true” species richness in the region that can be predicted by each richness estimator. The estimated standard error (*SE*) and CP associated with Fisher's original model are in parentheses

True value	Method	Average	Sample *SE*	Estimated *SE*	CP
ω = 0.1 *A* = 100 *S_A_* = 6,000	$\hat{\omega }$	0.1002	0.0046	0.0045	94.8
	*S*_*a*_	2,082.9	37.2	(22.1) 36.9	(74.8) 95
	${\hat{S}}_{A}$	6,002.8	124.3	(24.5) 123.9	(29.4) 94.8
	${\hat{S}}_{Chao1}$	2,956.1	91.6	82.9	0.0
	${\hat{S}}_{\mathrm{ACE}}$	2,923.5	74.4	62.9	0.0
	${\hat{S}}_{Jk1}$	2,872.8	56.0	39.7	0.0
ω = 0.05 *A* = 100 *S_A_* = 6,000	$\hat{\omega }$	0.0501	0.0022	0.0022	94.7
	*S*_*a*_	2,402.7	38.2	(22.2) 38	(75) 94.6
	${\hat{S}}_{A}$	5,999.1	105.4	(23.4) 106.2	(35) 95.1
	${\hat{S}}_{Chao1}$	3,195.9	85.9	76.8	0.0
	${\hat{S}}_{\mathrm{ACE}}$	3,122.1	66.8	54.6	0.0
	${\hat{S}}_{Jk1}$	3,154.5	54.8	38.8	0.0
ω = 0.01 *A* = 100 *S_A_* = 6,000	$\hat{\omega }$	0.01	4e‐04	4e‐04	95.1
	*S*_*a*_	3,006.2	39	(30) 38.7	(86.8) 94.4
	${\hat{S}}_{A}$	5,999.5	82.8	(24.9) 82.3	(45) 94.9
	${\hat{S}}_{Chao1}$	3,661.0	77.8	68.3	0.0
	${\hat{S}}_{\mathrm{ACE}}$	3,564.6	58.9	45.6	0.0
	${\hat{S}}_{Jk1}$	3,651.0	51.9	35.9	0.0
ω = 0.005 *A* = 100 *S_A_* = 6,000	$\hat{\omega }$	0.005	2e‐04	2e‐04	95
	*S*_*a*_	3,213.5	38.6	(31.3) 38.6	(88.6) 95.4
	${\hat{S}}_{A}$	6,001.2	75.7	(24.9) 75.9	(47.2) 95.2
	${\hat{S}}_{Chao1}$	3,822.7	74.8	65.6	0.0
	${\hat{S}}_{\mathrm{ACE}}$	3,729.6	57.2	43.5	0.0
	${\hat{S}}_{Jk1}$	3,816.8	50.6	34.7	0.0

For the simulated local species richness, our proposed variance calculation formula (Equation (3)) was asymptotically consistent or unbiased compared to Fisher's original variance calculation formula (Equation S10 in the Supporting Information), when comparing the estimated *SE* with the sample *SE* from the simulated data (Table 2 and Tables S1–S3). In contrast, the original Fisher's variance method presented remarkable biases (being much smaller) with respect to the “true” variance computed directly from the simulated data.

To extrapolate species richness over the entire region based on the simulated local data, estimated standard errors (*SE*s) using our area‐based method were always very close to the sample *SE*s computed directly from the simulated datasets (Table 2 and Tables S1–S3). In contrast, the estimated *SE* computed from original Fisher's method failed to provide a reasonable approximation of the sample *SE* for the entire region (Table 2 and Tables S1–S3), regardless of how the configuration (e.g., regional species number, regional area size, or parameter ω) for the simulations changed.

**Figure 2:**
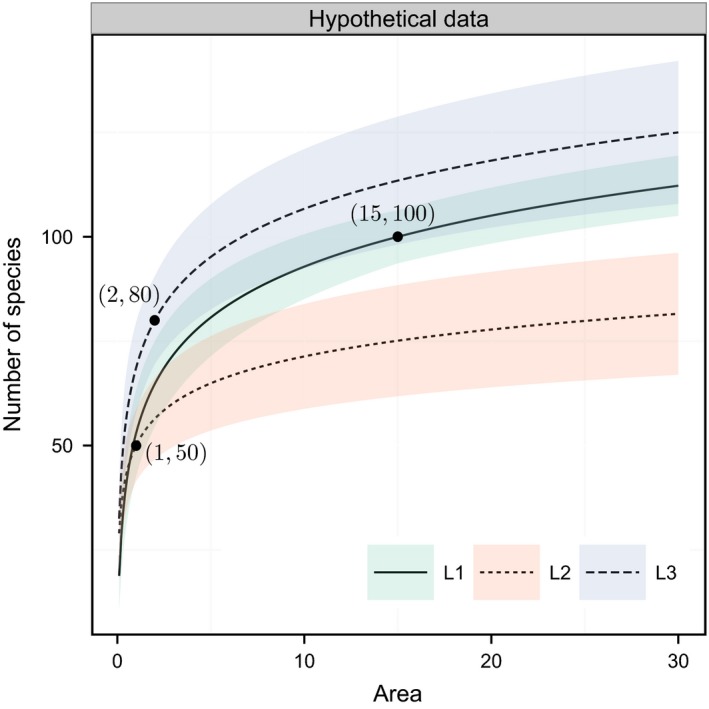
Fitting, rarefaction, and extrapolation of species richness in three hypothetical sites (L1, L2, and L3) from a hypothetical region *A *=* *30 using the proposed area‐based Fisher's alpha method. Site L1 has species number = 100, total individual number = 5,000, and sampling area size = 15; site L2 has species number = 50, total individual number = 2,000, and sampling area size = 1; and site L3 has species number = 80, total individual number = 2,000, and sampling area size = 2. Last, we assumed that the species abundance distributions (SADs) of all sites and the entire region are known a priori to follow the Fisher's logseries distribution. The 95% confidence intervals were calculated using the variance formula from Equation ((3)

Another theoretical example for performing area‐based rarefaction showed that the hypothetical sites, L1, L2, and L3, actually had different species richness levels at a given baseline area of 10 (any other local area or the entire region *A *=* *30 could be the baseline area; Figure 2): L3 was actually expected to have the highest species richness, followed by L1 and L2. Although there was some overlap between the 95% CIs for the species richness between these sites, site L3 was always expected to have an average species richness that was higher than the mean species richness for site L1 across the entire region (Figure 2). Moreover, even though both L2 and L3 had the same total sampled individual numbers and site L2 had a higher ratio of species richness to sampling area (50/1 = 50), the rarefaction curves consistently implied that L3 had higher species richness than L2 across the entire region (Figure 2).

When data were generated rather than from the area‐based logseries model (Tables S4–S6), the extrapolated richness estimated by the proposed method would be considerably overestimated in some cases. For example, the mean estimate 9705.1 by the proposed method is about as large as 1.5 times the true richness 6,000 for TPLN(0, 1.5) in Table S4; applying the proposed model to the data from TNBD with *k *=* *1 and ω = 0.01 or 1 led to the mean estimates 12,140.1 (Table S5) or 13,340 (Table S6), respectively. However, for TNBD with *k* becoming small, the mean estimates of the proposed method will gradually approach to the true richness as the area‐based logseries is derived from TNBD as *k* goes to zero. However, to avoid incurring the overestimation of species richness, conducting some goodness‐of‐fit tests (e.g., KS and χ^2^ tests) on the observed data should be necessary prior to using the proposed method.

**Figure 3:**
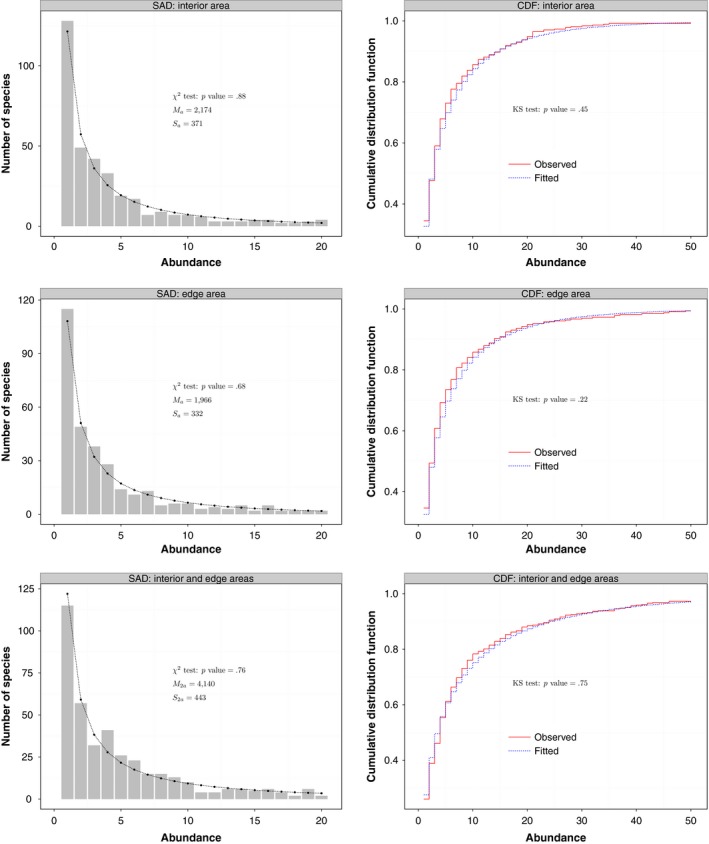
A comparison of the fitting performance of the proposed area‐based Fisher's alpha method on tree species from either interior, edge, or both areas of fragments of Brazilian Atlantic forests. We fit our area‐based model to two local areas (interior versus edge areas: the first four panels), both had the same local sampling area size of 1.2 ha; and the combined area had an area size of 2.4 ha (the last two panels)

The empirical datasets of tree species diversity in Brazilian Atlantic Forests fit very well, if not perfect, using our proposed area‐based logseries model. As can be seen, both the KS and χ^2^ tests gave *p* values that were much larger than the significance threshold of 0.05 for the empirical dataset (Figure 3). Additionally, the cumulative distribution functions (CDFs) between the observed data and fitted model were almost indistinguishable from each other for both the edge and interior empirical datasets and the combined data of both (Figure 3).

The proposed area‐based Fisher's alpha method estimated that species richness at a broader spatial scale, which combined all sampled plots from both edge and interior areas, was 456 with a 95% CI of 415–498, when using local plots from edge areas only (Table 3). The estimated species richness became 408 with a 95% CI of 369–447 when only sampled plots from interior areas were used (Table 3). The 95% CIs of richness at the 2‐time extrapolated spatial scale always encompassed the true observed species richness (443) reported in Magnago *et al*. (2014). In comparison, not all of the 95% CIs from the nonparametric estimators encompassed the true observed species richness (Table 3). This was particularly true for estimates using the edge local dataset only: The two nonparametric methods, Chao1 and ACE estimators, were found to have underestimated the true species richness at the 2‐time extrapolated spatial scale (Table 3).

**Table 3:** Empirical validation of the richness extrapolation or interpolation power using our proposed area‐based model from either interior, edge, or the combined augmented areas in fragments of Brazilian Atlantic forests. For richness extrapolation from each local dataset (interior or edge areas only; second and third columns), the local sampling area size was *a *=* *12 ha, and accordingly, the combination of both areas had an augmented area A* = 2.4 ha. The last column represents the interpolation of species richness from the augmented area (*a *=* *2.4 ha) for either interior or edge areas (A* = 1.2 ha). The proposed area‐based model with a single parameter, ω (the variance was estimated using Equation (3)), was compared with nonparametric methods, the relevant point estimation, and variance calculation methods, which are presented in the Supporting Information. The jackknife estimator was inapplicable here. The 95% confidence intervals for each estimator are in parentheses, where ¶ indicates that a log transformation (Chao 1987; Chiu *et al*. 2014) was applied to the confidence interval. Because richness interpolation by Hurlbert (1971) was an individual‐based method, different values were reported for the 1.2‐ha interior and edge areas (as they have different species frequency data) when performing richness interpolation from the augmented 2.4‐ha area. As a comparison, richness interpolation by Coleman (1981) is an area‐based method. Therefore, like our proposed area‐based method, it returned a single value for both interior and edge areas, given that both have the same sampling area sizes (1.2 ha)

	Brazilian Atlantic forests		
	Extrapolation		Interpolation
Methods	Interior areas only	Edge areas only	Interior+Edge combined areas
	(*M*_*a*_ * = *2,174, *S*_*a*_ = 371)	(*M*_*a*_ * = *1,966*, S*_*a*_ = 332)	(*M*_*a*_ = 4,140, *S*_*a*_ = 443)
Area‐based logseries	${\hat{S}}_{A*}=456\;\left(415,498\right)$ $\hat{\omega }=0.071\;\left(0.056,0.086\right)$	${\hat{S}}_{A^{*}}=408\;\left(369,447\right)$ $\hat{\omega }=0.070\;\left(0.054,0.086\right)$	${\hat{S}}_{A^{*}}=360\;\left(326,393\right)$ $\hat{\omega }=0.073\;\left(0.058,0.088\right)$
Chao1/Hurlbert	${\hat{S}}_{Chao1}=454\;\left(433,484\right)$ ^¶^	${\hat{S}}_{Chao1}=414\;\left(394,442\right)$ ^¶^	Interior: ${\hat{S}}_{\mathrm{Hurlbert}}^{\mathrm{Interior}}=368\;\left(358,379\right)$ Edge: ${\hat{S}}_{\mathrm{Hurlbert}}^{\mathrm{Edge}}=357\;\left(347,367\right)$
ACE/Coleman	${\hat{S}}_{\mathrm{ACE}}=451\;\left(429,481\right)$ ^¶^	${\hat{S}}_{\mathrm{ACE}}=405\;\left(384,434\right)$ ^¶^	${\hat{S}}_{\mathrm{Coleman}}=363\;\left(347,380\right)$

**Figure 4:**
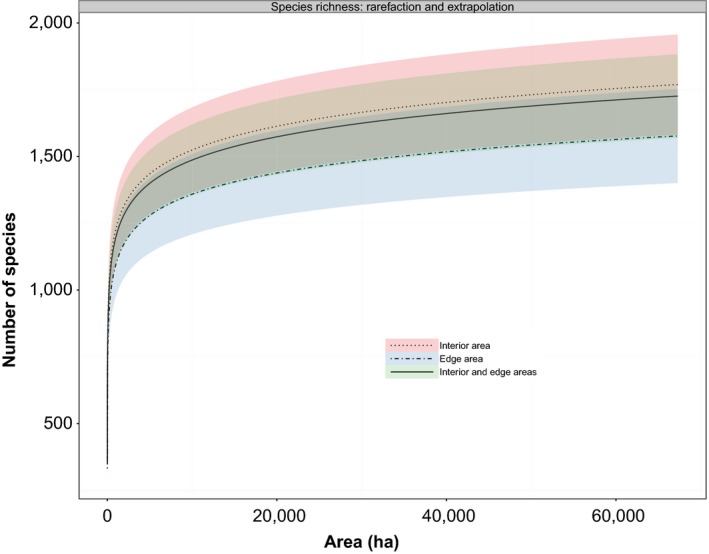
Rarefaction and extrapolation by the proposed area‐based Fisher's alpha method on tree species from either interior, edge, or the combined areas or fragments of Brazilian Atlantic forests (the entire region had an area size *A *=* *67,282.16 ha). The rarefaction/extrapolation curves were generated using the fitted area‐based models presented in Figure 3 (the fitted parameters are reported in Table 3). The 95% confidence intervals were calculated using the variance formula from Equation (3)

Extrapolation of regional species richness to the entire region (composed of all 12 fragments in the Brazilian Atlantic forests, please refer to Figure 1 in Magnago et al.'s paper) showed that the 95% CIs largely overlapped, regardless of whether species richness was extrapolated from the plots of the interior area only, the edge area only, or the combination of both areas (Figure 4). Species richness was estimated to be in a range 1,577–1,769 by the area‐based logseries model against a range 447–559 by the three nonparametric methods for the entire fragment region (*A *=* *67,282.16 ha), depending on the local dataset used (Figure 4; Table 4). Moreover, the 95% CIs by these estimates largely overlapped (Figure 4, Table 4). However, all three nonparametric methods predicted that regional species richness had much smaller values (Table 4). Furthermore, the 95% CIs by these estimators sometimes did not overlap when estimated from different local datasets (i.e., interior, edge, or the combined areas). For example, for the ACE estimator, the 95% CIs did not overlap between the case when local edge‐area data were used and the case when combined data from augmented areas were used (Table 4).

**Table 4:** Extrapolation of species richness for the entire region (area size *A *=* *67,282.16 ha) from either interior, edge, or combined augmented areas in fragments of Brazilian Atlantic Forests. The proposed area‐based model was compared to three nonparametric models, the relevant point estimation, and variance calculation methods of which are presented in the Supporting Information. The 95% confidence interval for each estimator is in parentheses, where ¶ indicates that a log transformation (Chao 1987; Chiu *et al*. 2014) was applied to the confidence interval

	Brazilian Atlantic Forests: Extrapolation of regional species richness		
	Interior areas only	Edge areas only	Interior+Edge combined areas
Methods	(*M*_*a*_ * *= 2,174, *S*_*a*_ * *= 371)	(*M*_*a*_ * *= 1,966, *S*_*a*_ * *= 332)	(*M*_*2a*_ * *= 4,140, *S*_*2a*_ * *= 443)
Area‐based	${\hat{S}}_{A}=1,769\;\left(1,581,1,956\right)$	${\hat{S}}_{A}=1,577\;\left(1,400,1,753\right)$	${\hat{S}}_{A}=1,726\;\left(1,569,1,882\right)$
Chao1	${\hat{S}}_{Chao1}=538\;\left(476,637\right)$ ^¶^	${\hat{S}}_{Chao1}=467\;\left(415,551\right)$ ^¶^	${\hat{S}}_{Chao1}=559\;\left(515,630\right)$ ^¶^
ACE	${\hat{S}}_{\mathrm{ACE}}=496\;\left(458,551\right)$ ^¶^	${\hat{S}}_{\mathrm{ACE}}=447\;\left(410,501\right)$ ^¶^	${\hat{S}}_{\mathrm{ACE}}=533\;\left(505,575\right)$ ^¶^
First‐order Jackknife	${\hat{S}}_{Jk1}=499\;\left(468,530\right)$	${\hat{S}}_{Jk1}=447\;\left(417,477\right)$	${\hat{S}}_{Jk1}=558\;\left(528,588\right)$

## 4 DISCUSSION

When predicting species richness or species extinctions, Fisher's alpha actually does not need to take abundance frequencies of species in the local sample into account, as shown in previous empirical studies (Gilbert *et al*. 2006; Slik *et al*. 2015). This is different from a perspective of nonparametric species richness estimation (Chao & Chiu 2016), which incorporated species abundance frequencies as data inputs to estimate species richness. However, whether the species frequencies are crucial to species richness estimation depends on what model is employed. Nevertheless, we can statistically prove that Fisher's alpha index either in Fisher *et al*. (1943)'s original paper or in our study does not rely on sampling frequency information at all. This is because, as demonstrated in the full maximum‐likelihood equation (Equations (1) and (1)), Fisher's alpha needs very parsimonious information when predicting species diversity, including the observed species richness and observed individual number. These two quantities are sufficient statistics for inferring Fisher's alpha parameter in both Fisher's paper and our area‐based model. In contrast, most existing richness estimators (particularly nonparametric methods) ask for species abundance frequency data as data inputs which sometimes may be unavailable (e.g., in an imperfectly sampled case).

Previous studies argued that when abundance‐rank plots are used and the abundance of each species is log‐transformed, the fitted curves for both geometric series and logseries models should be indistinguishable (Taylor *et al*. 1976; Fattorini 2005). However, we proved that Fisher's logseries was the most extreme scenario derived from the TNBD in predicting rare species and predicting maximal numbers of singleton and doubleton species that were always higher than any other TNBD‐derived models, including the geometric series model (when aggregation parameter *k *=* *1, see the detailed derivation from Theorem 2 in the Supporting Information). Moreover, our study showed that even though the geometric series model might be very suitable for characterizing extremely uneven SADs (Magurran 2004; Fattorini 2005), it did not perform well in predicting rare species richness. This is simply because it cannot predict a number of rare species (especially for singleton and doubleton species) as high as Fisher's logseries model. It can be mathematically proven that the proposed area‐based logseries model, derived from the TNBD, predicts the highest number of rare species (please refer to Theorem 3 in the Supporting Information).

If the area‐based logseries model is the basis of observed data, our model is extremely powerful, because species richness can be extrapolated at a regional scale, the spatial extent of which is much larger than that of local sampling sites (Hubbell 2015; Slik *et al*. 2015). As shown in the simulation tests from Table 2 and additional tables in the Supporting Information, the ratio of regional area size *A* to local sampling area *a* can be a very large value. In contrast, previous nonparametric methods, like the Chao1 estimator, would not be applicable, as the extrapolation range of these nonparametric statistical methods is very small (typically 2–3 times larger than the local area size) (Chao *et al*. 2016). Moreover, as expected, the underestimation problem becomes worse when the regional area size is larger (Table 2 and Tables S1–S3) (Chao & Chiu 2016; Chao *et al*. 2016). Therefore, when the spatial grain of the extrapolation becomes larger than those for local samples, nonparametric estimators should be carefully used, and one should be aware of the considerable underestimation of species richness if the logseries model fits the surveyed data very well. In this case, Fisher's alpha and the proposed area‐based version are recommended (Hubbell 2015; Slik *et al*. 2015). As a rule of thumb, if the ratio of the numbers of singleton to doubleton species in the surveyed species frequency data has a numeric value close to 2 (Figure 1), Fisher's logseries model is very likely. Moreover, goodness‐of‐fit tests can be conducted to further confirm this.

Nearly, all previous methods on the rarefaction and extrapolation of species richness believed that species richness would be meaningful and comparable for different communities as long as the number of individuals was interpolated or extrapolated to the same baseline value (Hurlbert 1971; Heck *et al*. 1975; Soetaert & Heip 1990; Gotelli & Colwell 2001). However, in addition to the individual number, our study also revealed that the sampling area size is important when comparing different communities. As demonstrated in Figure 2, even though hypothetical sites L2 and L3 had the same number of species individuals (=2,000), their species richness status could not be compared, because their sampling area sizes differed. After controlling for the sampling area size, it was consistently found that site L3 had higher species richness than L2.

We generated 36 combinations of results from the numerical tests (Tables S1–S3) to validate the estimation power and accuracy of regional species richness using the proposed area‐based model. As a comparison, all three nonparametric methods largely underestimated the regional species richness when sample data follow the proposed area‐based logseries model (Table 2 and Tables S1–S3). Moreover, through the empirical test on the tree diversity data from Brazilian forest fragments, when local sampling plots from edge areas were used, all nonparametric methods were found to underestimate the true species richness (even some of their 95% CIs failed to encompass the true value) for the augmented 2.4‐ha sampling area (Table 3). In contrast, the proposed model accurately predicted species richness for which the 95% CIs encompassed the true value, regardless of which local data were used. The situations for species richness interpolation, from the augmented area (2.4 ha) to either the interior or edge area, were also similar (Table 3). Therefore, nonparametric methods tend to underestimate true species richness in both numerical and empirical tests, especially when data are likely from the logseries model.

In the numerical simulation, the sample *SE* was always underestimated by the estimated *SE* (Table 2 and Tables S1–S3) calculated using the Fisher's original variance computational formula (Equation S10 in the Supporting Information). The underestimation was aggravated in the regional species richness estimation (${\hat{S}}_{A}$) (Table 2 and Tables S1–S3). The underestimation of sample *SE* is due to the fact that Fisher's original variance calculation formula was derived by fixing the number of individuals in the sample, while ignoring the sampling uncertainty caused by the difference in the number of simulated individuals in the local sample in different simulation rounds. Moreover, when the regional area is sufficiently large, the variance (or estimated *SE*) calculated using Fisher's original formula reaches an upper limit, which explains why the underestimation of sample *SE* was worse when estimating regional species richness (see the theoretical proof in the Supporting Information). By comparison, our proposed variance estimator (Equation (3)) can account for this simulation uncertainty by recognizing the fact that the species observed in sample *a* (their number was *S*_*a*_) are a part of those from the larger regional area *A*. This means that *S*_*a*_ follows a binomial distribution with total species number *S*_*A*_ and occurrence probability $\alpha_{A}/\alpha_{a}$ (see the term ${\left(\alpha_{A}/\alpha_{a}\right)}^{S_{a}}{\left(1-\alpha_{A}/\alpha_{a}\right)}^{S_{A}-S_{a}}$ from the likelihood function in Equation (1)).

The proposed area‐based model is statistically consistent, as indicated by two observations: (1) the estimated species richness for the augmented 2.4‐ha areas from either edge or interior areas was very close (Table 3); and (2) the estimated regional species richness for the entire region was very similar from different local datasets (edge, interior, or combined edge and interior areas) (Table 4; Figure 4). Other than these, the corresponding 95% CIs by these estimates largely overlapped (Tables 3, 4; Figure 4). These results demonstrated that the proposed area‐based model could consistently estimate regional species richness. This is expected, as species richness in augmented areas or the regional species richness over the 12 fragments estimated from local areas (edge, interior, or combined edge and interior areas) is a fixed value, even though its true value is yet unknown for the entire region (but richness in the augmented areas was known to be 443).

As the proposed area‐based logseries model is sensitive to the prediction of rare species in comparison with nonparametric methods, using the proposed method can lead to overestimation of species richness when data are not from the assumed model (Tables S5–S6). As a caveat, to determine whether or not the proposed model can be applied to the observed data, conducting some goodness‐of‐fit tests on the data is a very crucial step to avoid incurring the mentioned issue.

The goodness‐of‐fit statistic is critical for comparing performances of alternative ecological models (Waller *et al*. 2003). Currently, the nonparametric χ^2^ and KS tests are widely used in fitting theoretical probabilistic models to empirical SADs. However, these statistics should be used with caution. For example, the KS test was primarily developed for continuous probability models; thus, when applying it to test the goodness of fit of discrete probability models, some adjustments are required. Because species abundance is a standard discrete variable, a step function should be used (Arnold & Emerson 2011) to characterize CDFs of both empirical data and the fitted SAD models as in Figure 3 in our study. Consequently, it would be misleading to use smooth and continuous CDFs to characterize species abundances, which will increase Type II error. Last, the *p* value of the test should be adjusted using some techniques, like the Monte Carlo method (Arnold & Emerson 2011).

Finally, other than Magnago *et al*. (2014)'s species frequency count data used in our study (Table 1), the 2:1 ratio between singleton and doubleton species numbers actually is prevailingly reported in much of the other empirical literature (Norden *et al*. 2009; Longino & Colwell 2011; Colwell *et al*. 2012; Slik *et al*. 2015). All these empirical examples show that Fisher's logseries is a very important parametric model for fitting empirical species abundance data in ecology. Correspondingly, this 2:1 ratio can be a very good proxy to determine whether Fisher's logseries model (and our area‐based model, of course) should be applied in empirical settings. This ratio will be highly effective for quickly determining the applicability of Fisher's logseries, particularly when complete species frequency data are sometimes unavailable from field sampling, and consequently, goodness‐of‐fit statistics like the KS or χ^2^ tests cannot be used.

In conclusion, our study developed a general area‐based Fisher's alpha diversity model and derived an asymptotically unbiased variance formula, allowing it to perform both rarefaction and extrapolation of species richness so as to compare local species diversity between local samples with varying area sizes (and individual numbers as well) and predict the regional species richness. For future prospects, the present model has the potential to serve as a fundamental one when doing parametric estimation of species richness, given that our model is data parsimonious and Fisher's logseries has been broadly observed and applied in many ecological communities worldwide (Volkov *et al*. 2003, 2005; Gilbert *et al*. 2006; Norden *et al*. 2009; Longino & Colwell 2011; Colwell *et al*. 2012; Magnago *et al*. 2014; Hubbell 2015; Slik *et al*. 2015).

## AUTHORS CONTRIBUTION

Y.C. designed the study, conducted the analyses, and interpreted the results. T.J.S. derived the theoretical results and programmed the script. Both authors wrote and reviewed the manuscript.
