# Supplementary material for: Rarefaction and extrapolation of species richness using an area‐based Fisher's logseries
Source: Ecol Evol. 2017 Oct 23;7(23):10066–78. doi: 10.1002/ece3.3509 (PMC5723611; doi:10.1002/ece3.3509)
Supplement: Supplementary file 1 [file ECE3-7-10066-s001.pdf]

Table S1: Simulation and estimation summarized from all the configurations when total regional species richness S is fixed to 500.

True value	Method	Average	Sample SE	Estimated SE	CP
$\omega = 0.1$	$\hat{\omega}$	0.102	0.0161	0.016	95.2
$A = 100$	S_a	173.4	10.8	(6.4) 10.6	(75.3) 94.6
$S = 500$	$\hat{S}_A$	501	36.5	(7.1) 35.9	(29.2) 94.7
	$\hat{S}_{Chao1}$	249.1	28.2	24.9	0.1
	$\hat{S}_{ACE}$	244.3	21.8	18.3	0.0
	$\hat{S}_{Jk1}$	239.1	16.3	11.4	0.0
$\omega = 0.05$	$\hat{\omega}$	0.0511	0.008	0.0078	95.4
$A = 100$	S_a	200.3	11.1	(6.4) 11	(74.7) 94.4
$S = 500$	$\hat{S}_A$	501.1	31.1	(6.8) 30.8	(33.6) 94.6
	$\hat{S}_{Chao1}$	269.5	26.0	23.2	0.0
	$\hat{S}_{ACE}$	261.1	19.3	15.9	0.0
	$\hat{S}_{Jk1}$	263.0	15.9	11.2	0.0
$\omega = 0.01$	$\hat{\omega}$	0.0102	0.0016	0.0016	95.2
$A = 100$	S_a	250.6	11.1	(7.8) 11.2	(82.6) 95.3
$S = 500$	$\hat{S}_A$	500.4	23.6	(6.9) 23.8	(42.4) 95.1
	$\hat{S}_{Chao1}$	307.7	23.7	20.6	0.1
	$\hat{S}_{ACE}$	297.8	17.3	13.3	0.0
	$\hat{S}_{Jk1}$	304.3	15.0	10.3	0.0
$\omega = 0.005$	$\hat{\omega}$	0.0051	8e-04	8e-04	94.8
$A = 100$	S_a	267.7	11	(9) 11.1	(89.9) 95.4
$S = 500$	$\hat{S}_A$	499.9	21.8	(7.2) 21.9	(48.5) 95.2
	$\hat{S}_{Chao1}$	321.3	23.3	20.0	0.4
	$\hat{S}_{ACE}$	311.3	16.7	12.7	0.0
	$\hat{S}_{Jk1}$	317.9	14.6	10.0	0.0

True value	Method	Average	Sample SE	Estimated SE	CP
$\omega = 0.1$	$\hat{\omega}$	0.1029	0.0191	0.0186	95.4
$A = 1000$	S_a	130.1	9.7	(5.5) 9.8	(74.1) 95
$S = 500$	$\hat{S}_A$	502	45.1	(6.1) 45.3	(21.2) 95
	$\hat{S}_{Chao1}$	188.2	25.2	22.2	0.0
	$\hat{S}_{ACE}$	183.5	19.1	15.9	0.0
	$\hat{S}_{Jk1}$	179.4	14.5	9.9	0.0
$\omega = 0.05$	$\hat{\omega}$	0.0513	0.0092	0.0089	95.2
$A = 1000$	S_a	153.6	10.5	(5.6) 10.3	(70.8) 94.7
$S = 500$	$\hat{S}_A$	501.2	39.1	(5.9) 38.5	(23.9) 94.5
	$\hat{S}_{Chao1}$	207.5	24.6	20.7	0.0
	$\hat{S}_{ACE}$	200.5	18.4	14.0	0.0
	$\hat{S}_{Jk1}$	201.6	15.0	9.8	0.0
$\omega = 0.01$	$\hat{\omega}$	0.0103	0.0018	0.0018	95.4
$A = 1000$	S_a	200.3	11	(6.8) 11	(76.8) 94.9
$S = 500$	$\hat{S}_A$	500.6	29.8	(5.9) 29.6	(30.5) 95
	$\hat{S}_{Chao1}$	246.9	22.2	18.9	0.0
	$\hat{S}_{ACE}$	238.3	16.5	11.9	0.0
	$\hat{S}_{Jk1}$	243.3	14.6	9.2	0.0
$\omega = 0.005$	$\hat{\omega}$	0.0051	9e-04	9e-04	94.8
$A = 1000$	S_a	217.3	11.1	(8.1) 11.1	(84.9) 94.9
$S = 500$	$\hat{S}_A$	500.3	26.9	(6.2) 27.2	(34.8) 95
	$\hat{S}_{Chao1}$	261.4	22.3	18.4	0.1
	$\hat{S}_{ACE}$	252.8	16.2	11.5	0.0
	$\hat{S}_{Jk1}$	258.0	14.4	9.0	0.0

True value	Method	Average	Sample SE	Estimated SE	CP
$\omega = 0.1$	$\hat{\omega}$	0.1034	0.0219	0.0209	95
$A = 10000$	S_a	104	8.9	(4.9) 9.1	(73.4) 95.4
$S = 500$	$\hat{S}_A$	502.8	53.1	(5.5) 53.2	(15.9) 95
	$\hat{S}_{Chao1}$	151.3	23.2	20.2	0.0
	$\hat{S}_{ACE}$	147.1	17.6	14.3	0.0
	$\hat{S}_{Jk1}$	143.5	13.3	8.9	0.0
$\omega = 0.05$	$\hat{\omega}$	0.0517	0.0102	0.01	95.4
$A = 10000$	S_a	124.6	9.8	(5.1) 9.7	(68.7) 94.2
$S = 500$	$\hat{S}_A$	502	45.7	(5.3) 45.1	(17.8) 94.2
	$\hat{S}_{Chao1}$	169.3	23.1	19.1	0.0
	$\hat{S}_{ACE}$	162.9	16.9	12.7	0.0
	$\hat{S}_{Jk1}$	163.6	14.0	8.8	0.0
$\omega = 0.01$	$\hat{\omega}$	0.0103	0.002	0.0019	95.2
$A = 10000$	S_a	166.8	10.6	(6.1) 10.5	(73.6) 94.5
$S = 500$	$\hat{S}_A$	500.5	34.7	(5.3) 34.4	(23.1) 94.7
	$\hat{S}_{Chao1}$	206.3	21.7	17.5	0.0
	$\hat{S}_{ACE}$	198.7	15.4	10.9	0.0
	$\hat{S}_{Jk1}$	202.7	13.9	8.4	0.0
$\omega = 0.005$	$\hat{\omega}$	0.0052	0.001	0.001	95.4
$A = 10000$	S_a	182.5	10.9	(7.4) 10.7	(82.1) 94.6
$S = 500$	$\hat{S}_A$	499.6	33.2	(5.5) 31.6	(25.9) 94.6
	$\hat{S}_{Chao1}$	220.3	21.5	17.2	0.1
	$\hat{S}_{ACE}$	212.5	15.7	10.6	0.0
	$\hat{S}_{Jk1}$	216.7	14.1	8.2	0.0

True value	Method	Average	Sample SE	Estimated SE	CP
$\omega = 0.1$	$\hat{\omega}$	0.1046	0.0236	0.0226	95
$A = 50000$	S_a	91.4	8.7	(4.6) 8.6	(70.2) 94.6
$S = 500$	$\hat{S}_A$	505.2	58.3	(5.2) 58.4	(14) 94.9
	$\hat{S}_{Chao1}$	133.4	22.4	19.2	0.0
	$\hat{S}_{ACE}$	129.3	16.8	13.4	0.0
	$\hat{S}_{Jk1}$	126.1	12.9	8.3	0.0
$\omega = 0.05$	$\hat{\omega}$	0.0521	0.011	0.0107	95.4
$A = 50000$	S_a	110.2	9.2	(4.8) 9.3	(68.5) 94.8
$S = 500$	$\hat{S}_A$	503.3	48.6	(5) 49.3	(16) 95.3
	$\hat{S}_{Chao1}$	150.2	22.1	18.3	0.0
	$\hat{S}_{ACE}$	144.1	15.7	11.9	0.0
	$\hat{S}_{Jk1}$	144.7	13.0	8.3	0.0
$\omega = 0.01$	$\hat{\omega}$	0.0104	0.0022	0.0021	94.9
$A = 50000$	S_a	149.8	10.3	(5.7) 10.2	(72) 94.7
$S = 500$	$\hat{S}_A$	502.1	37.9	(5) 37.5	(20.5) 94.9
	$\hat{S}_{Chao1}$	185.6	21.0	16.8	0.1
	$\hat{S}_{ACE}$	178.3	14.9	10.3	0.0
	$\hat{S}_{Jk1}$	181.9	13.5	8.0	0.0
$\omega = 0.005$	$\hat{\omega}$	0.0052	0.0011	0.001	95.2
$A = 50000$	S_a	164.7	10.4	(7) 10.5	(80.7) 94.7
$S = 500$	$\hat{S}_A$	499.7	43.1	(5.2) 34.3	(23.2) 94.5
	$\hat{S}_{Chao1}$	199.2	20.0	16.5	0.0
	$\hat{S}_{ACE}$	192.0	14.6	10.1	0.0
	$\hat{S}_{Jk1}$	195.7	13.3	7.8	0.0

Table S2: Simulation and estimation summarized from all the configurations when total regional species richness S is fixed to 2000.

True value	Method	Average	Sample SE	Estimated SE	CP
$\omega = 0.1$	$\hat{\omega}$	0.1003	0.0077	0.0078	95.6
$A = 100$	S_a	694.4	21.4	(12.8) 21.3	(75.1) 94.9
$S = 2000$	$\hat{S}_A$	2001	71.5	(14.2) 71.5	(30.3) 94.9
	$\hat{S}_{Chao1}$	987.5	53.5	48.2	0.0
	$\hat{S}_{ACE}$	974.9	42.9	36.3	0.0
	$\hat{S}_{Jk1}$	957.5	32.2	22.9	0.0
$\omega = 0.05$	$\hat{\omega}$	0.0503	0.0039	0.0038	94.2
$A = 100$	S_a	801.1	21.9	(12.8) 21.9	(75.3) 94.8
$S = 2000$	$\hat{S}_A$	2001.2	61.1	(13.5) 61.4	(34.4) 94.9
	$\hat{S}_{Chao1}$	1068.2	49.8	44.8	0.0
	$\hat{S}_{ACE}$	1041.7	38.5	31.6	0.0
	$\hat{S}_{Jk1}$	1051.9	31.5	22.4	0.0
$\omega = 0.01$	$\hat{\omega}$	0.0101	8e-04	8e-04	94.6
$A = 100$	S_a	1001.8	22.2	(17.3) 22.4	(87.7) 95.2
$S = 2000$	$\hat{S}_A$	1999.6	47.5	(14.4) 47.5	(44.1) 95
	$\hat{S}_{Chao1}$	1221.4	45.2	39.7	0.0
	$\hat{S}_{ACE}$	1188.0	33.7	26.3	0.0
	$\hat{S}_{Jk1}$	1216.5	29.8	20.7	0.0
$\omega = 0.005$	$\hat{\omega}$	0.005	4e-04	4e-04	94.8
$A = 100$	S_a	1070.5	22.5	(18.1) 22.3	(88.3) 94.8
$S = 2000$	$\hat{S}_A$	1998.9	44	(14.4) 43.8	(49) 95
	$\hat{S}_{Chao1}$	1274.6	44.2	38.2	0.0
	$\hat{S}_{ACE}$	1242.1	33.6	25.1	0.0
	$\hat{S}_{Jk1}$	1270.9	29.7	20.0	0.0

True value	Method	Average	Sample SE	Estimated SE	CP
$\omega = 0.1$	$\hat{\omega}$	0.1008	0.0093	0.0091	94.6
$A = 1000$	S_a	520.3	20	(11.1) 19.6	(72) 94.2
$S = 2000$	$\hat{S}_A$	2001.6	92.3	(12.3) 90	(21.8) 94.2
	$\hat{S}_{Chao1}$	741.1	48.5	41.9	0.0
	$\hat{S}_{ACE}$	730.9	38.5	31.5	0.0
	$\hat{S}_{Jk1}$	717.6	29.7	19.9	0.0
$\omega = 0.05$	$\hat{\omega}$	0.0504	0.0044	0.0044	95.2
$A = 1000$	S_a	614.9	20.7	(11.2) 20.6	(71) 95
$S = 2000$	$\hat{S}_A$	2002.1	76.6	(11.8) 76.7	(24.6) 94.8
	$\hat{S}_{Chao1}$	821.3	45.4	39.5	0.0
	$\hat{S}_{ACE}$	800.3	35.5	27.8	0.0
	$\hat{S}_{Jk1}$	807.6	29.4	19.6	0.0
$\omega = 0.01$	$\hat{\omega}$	0.0101	9e-04	9e-04	95
$A = 1000$	S_a	802	22	(15.4) 21.9	(82.3) 94.8
$S = 2000$	$\hat{S}_A$	2001	58.9	(12.4) 59	(31.1) 95.3
	$\hat{S}_{Chao1}$	979.9	43.3	35.9	0.0
	$\hat{S}_{ACE}$	952.0	32.7	23.6	0.0
	$\hat{S}_{Jk1}$	974.3	29.3	18.5	0.0
$\omega = 0.005$	$\hat{\omega}$	0.005	4e-04	4e-04	94.8
$A = 1000$	S_a	869.3	22.3	(16.3) 22.2	(84.5) 94.8
$S = 2000$	$\hat{S}_A$	2000.9	54.6	(12.4) 54.3	(34.9) 94.8
	$\hat{S}_{Chao1}$	1035.7	42.0	34.5	0.0
	$\hat{S}_{ACE}$	1009.1	32.7	22.6	0.0
	$\hat{S}_{Jk1}$	1032.2	29.4	18.0	0.0

True value	Method	Average	Sample SE	Estimated SE	CP
$\omega = 0.1$	$\hat{\omega}$	0.101	0.0102	0.0102	95.2
$A = 10000$	S_a	416.3	18.3	(9.9) 18.2	(71.1) 94.5
$S = 2000$	$\hat{S}_A$	2002.9	105.7	(11) 105.6	(15.1) 95.2
	$\hat{S}_{Chao1}$	593.3	43.7	37.6	0.0
	$\hat{S}_{ACE}$	584.7	35.2	28.1	0.0
	$\hat{S}_{Jk1}$	574.0	27.3	17.7	0.0
$\omega = 0.05$	$\hat{\omega}$	0.0503	0.0049	0.0049	95.4
$A = 10000$	S_a	498.4	19.4	(10.1) 19.3	(68.9) 95
$S = 2000$	$\hat{S}_A$	1999.9	89.5	(10.7) 89.6	(18.9) 94.5
	$\hat{S}_{Chao1}$	664.4	41.5	35.4	0.0
	$\hat{S}_{ACE}$	647.6	32.5	24.9	0.0
	$\hat{S}_{Jk1}$	654.0	27.5	17.6	0.0
$\omega = 0.01$	$\hat{\omega}$	0.0101	9e-04	9e-04	95.2
$A = 10000$	S_a	668	21.1	(14) 21.1	(80.9) 94.9
$S = 2000$	$\hat{S}_A$	2000.7	68.8	(11) 68.7	(24.4) 94.9
	$\hat{S}_{Chao1}$	816.1	39.3	32.8	0.0
	$\hat{S}_{ACE}$	792.8	30.6	21.6	0.0
	$\hat{S}_{Jk1}$	811.3	27.7	16.9	0.0
$\omega = 0.005$	$\hat{\omega}$	0.005	5e-04	5e-04	95.2
$A = 10000$	S_a	731.6	21.5	(14.9) 21.5	(82.5) 94.9
$S = 2000$	$\hat{S}_A$	2002.5	62.7	(11.1) 63.2	(27.4) 95.2
	$\hat{S}_{Chao1}$	872.8	39.3	31.9	0.0
	$\hat{S}_{ACE}$	849.6	30.5	20.8	0.0
	$\hat{S}_{Jk1}$	868.9	27.7	16.6	0.0

True value	Method	Average	Sample SE	Estimated SE	CP
$\omega = 0.1$	$\hat{\omega}$	0.101	0.0112	0.0109	94.5
$A = 50000$	S_a	365.5	17.7	(9.3) 17.3	(70.7) 94
$S = 2000$	$\hat{S}_A$	2004.4	117.6	(10.3) 115.3	(13) 94.3
	$\hat{S}_{Chao1}$	522.3	41.6	35.5	0.0
	$\hat{S}_{ACE}$	514.3	33.7	26.5	0.0
	$\hat{S}_{Jk1}$	504.2	26.3	16.6	0.0
$\omega = 0.05$	$\hat{\omega}$	0.0505	0.0052	0.0052	95.5
$A = 50000$	S_a	440.8	18.7	(9.5) 18.5	(67.5) 94.7
$S = 2000$	$\hat{S}_A$	2003.9	97.9	(10) 97.8	(15.4) 95.2
	$\hat{S}_{Chao1}$	589.4	39.5	33.6	0.0
	$\hat{S}_{ACE}$	573.6	31.2	23.5	0.0
	$\hat{S}_{Jk1}$	578.8	26.4	16.6	0.0
$\omega = 0.01$	$\hat{\omega}$	0.0101	0.001	0.001	95.1
$A = 50000$	S_a	598.7	20.2	(13.1) 20.5	(79.8) 94.8
$S = 2000$	$\hat{S}_A$	2002.4	73.9	(10.3) 74.8	(22.5) 95.3
	$\hat{S}_{Chao1}$	731.3	37.8	31.0	0.0
	$\hat{S}_{ACE}$	710.5	29.4	20.4	0.0
	$\hat{S}_{Jk1}$	727.0	26.5	16.0	0.0
$\omega = 0.005$	$\hat{\omega}$	0.005	5e-04	5e-04	94.8
$A = 50000$	S_a	657.9	21	(14.2) 21	(82.1) 94.6
$S = 2000$	$\hat{S}_A$	2000	68.1	(10.4) 68.6	(23) 95.3
	$\hat{S}_{Chao1}$	785.4	37.5	30.4	0.0
	$\hat{S}_{ACE}$	764.4	29.3	19.8	0.0
	$\hat{S}_{Jk1}$	781.5	26.8	15.7	0.0

Table S3: Simulation and estimation summarized from all the configurations when total regional species richness S is fixed to 6000.

True value	Method	Average	Sample SE	Estimated SE	CP
$\omega = 0.1$	$\hat{\omega}$	0.1002	0.0046	0.0045	94.8
$A = 100$	S_a	2082.9	37.2	(22.1) 36.9	(74.8) 95
$S = 6000$	$\hat{S}_A$	6002.8	124.3	(24.5) 123.9	(29.4) 94.8
	$\hat{S}_{Chao1}$	2956.1	91.6	82.9	0.0
	$\hat{S}_{ACE}$	2923.5	74.4	62.9	0.0
	$\hat{S}_{Jk1}$	2872.8	56.0	39.7	0.0
$\omega = 0.05$	$\hat{\omega}$	0.0501	0.0022	0.0022	94.7
$A = 100$	S_a	2402.7	38.2	(22.2) 38	(75) 94.6
$S = 6000$	$\hat{S}_A$	5999.1	105.4	(23.4) 106.2	(35) 95.1
	$\hat{S}_{Chao1}$	3195.9	85.9	76.8	0.0
	$\hat{S}_{ACE}$	3122.1	66.8	54.6	0.0
	$\hat{S}_{Jk1}$	3154.5	54.8	38.8	0.0
$\omega = 0.01$	$\hat{\omega}$	0.01	4e-04	4e-04	95.1
$A = 100$	S_a	3006.2	39	(30) 38.7	(86.8) 94.4
$S = 6000$	$\hat{S}_A$	5999.5	82.8	(24.9) 82.3	(45) 94.9
	$\hat{S}_{Chao1}$	3661.0	77.8	68.3	0.0
	$\hat{S}_{ACE}$	3564.6	58.9	45.6	0.0
	$\hat{S}_{Jk1}$	3651.0	51.9	35.9	0.0
$\omega = 0.005$	$\hat{\omega}$	0.005	2e-04	2e-04	95
$A = 100$	S_a	3213.5	38.6	(31.3) 38.6	(88.6) 95.4
$S = 6000$	$\hat{S}_A$	6001.2	75.7	(24.9) 75.9	(47.2) 95.2
	$\hat{S}_{Chao1}$	3822.7	74.8	65.6	0.0
	$\hat{S}_{ACE}$	3729.6	57.2	43.5	0.0
	$\hat{S}_{Jk1}$	3816.8	50.6	34.7	0.0

True value	Method	Average	Sample SE	Estimated SE	CP
$\omega = 0.1$	$\hat{\omega}$	0.1002	0.0052	0.0052	95.2
$A = 1000$	S_a	1561.6	33.9	(19.2) 34	(72.6) 95.3
$S = 6000$	$\hat{S}_A$	5999.4	156.2	(21.2) 155.7	(21.5) 95.1
	$\hat{S}_{Chao1}$	2216.0	82.2	71.7	0.0
	$\hat{S}_{ACE}$	2191.0	66.4	54.4	0.0
	$\hat{S}_{Jk1}$	2153.3	50.7	34.4	0.0
$\omega = 0.05$	$\hat{\omega}$	0.0501	0.0025	0.0025	95.1
$A = 1000$	S_a	1844.3	35.4	(19.4) 35.7	(71.6) 95.2
$S = 6000$	$\hat{S}_A$	6000.9	132.6	(20.5) 132.7	(23.8) 94.9
	$\hat{S}_{Chao1}$	2452.9	76.5	67.3	0.0
	$\hat{S}_{ACE}$	2396.0	60.6	47.8	0.0
	$\hat{S}_{Jk1}$	2421.1	50.5	34.0	0.0
$\omega = 0.01$	$\hat{\omega}$	0.01	5e-04	5e-04	95.3
$A = 1000$	S_a	2406.2	38.1	(26.9) 38	(83.2) 94.6
$S = 6000$	$\hat{S}_A$	6004.1	102.4	(21.5) 102.1	(32.2) 94.6
	$\hat{S}_{Chao1}$	2930.6	72.0	61.1	0.0
	$\hat{S}_{ACE}$	2853.3	55.9	40.8	0.0
	$\hat{S}_{Jk1}$	2922.4	50.2	32.1	0.0
$\omega = 0.005$	$\hat{\omega}$	0.005	3e-04	3e-04	94.5
$A = 1000$	S_a	2607.2	38.4	(28.2) 38.4	(84.5) 94.4
$S = 6000$	$\hat{S}_A$	6001.4	94.2	(21.5) 94	(34.9) 94.9
	$\hat{S}_{Chao1}$	3103.2	72.1	59.3	0.0
	$\hat{S}_{ACE}$	3025.9	55.6	39.2	0.0
	$\hat{S}_{Jk1}$	3096.7	50.0	31.3	0.0

True value	Method	Average	Sample SE	Estimated SE	CP
$\omega = 0.1$	$\hat{\omega}$	0.1004	0.0058	0.0059	95.2
$A = 10000$	S_a	1249.1	31.9	(17.1) 31.4	(70.2) 94.7
$S = 6000$	$\hat{S}_A$	6002	183.7	(19) 182.6	(15.4) 95.2
	$\hat{S}_{Chao1}$	1773.6	73.7	64.3	0.0
	$\hat{S}_{ACE}$	1753.6	60.6	48.7	0.0
	$\hat{S}_{Jk1}$	1722.8	47.3	30.8	0.0
$\omega = 0.05$	$\hat{\omega}$	0.0501	0.0028	0.0028	94.7
$A = 10000$	S_a	1497.6	33.9	(17.5) 33.5	(68.9) 94.5
$S = 6000$	$\hat{S}_A$	6006.2	159	(18.5) 155.1	(17.7) 94.2
	$\hat{S}_{Chao1}$	1993.2	70.9	60.8	0.0
	$\hat{S}_{ACE}$	1946.7	56.1	43.1	0.0
	$\hat{S}_{Jk1}$	1966.4	47.7	30.6	0.0
$\omega = 0.01$	$\hat{\omega}$	0.01	5e-04	5e-04	94.9
$A = 10000$	S_a	2004.5	35.9	(24.5) 36.5	(81.9) 95.3
$S = 6000$	$\hat{S}_A$	6002	117.1	(19.2) 118.9	(25.6) 95.5
	$\hat{S}_{Chao1}$	2442.8	67.9	55.9	0.0
	$\hat{S}_{ACE}$	2377.7	52.9	37.3	0.0
	$\hat{S}_{Jk1}$	2434.8	47.5	29.3	0.0
$\omega = 0.005$	$\hat{\omega}$	0.005	3e-04	3e-04	94.2
$A = 10000$	S_a	2193.9	36.9	(25.9) 37.3	(83.3) 95.4
$S = 6000$	$\hat{S}_A$	6002.5	108.9	(19.2) 109.3	(27.1) 95.1
	$\hat{S}_{Chao1}$	2611.2	66.6	54.4	0.0
	$\hat{S}_{ACE}$	2546.0	52.4	35.9	0.0
	$\hat{S}_{Jk1}$	2605.6	47.7	28.7	0.0

True value	Method	Average	Sample SE	Estimated SE	CP
$\omega = 0.1$	$\hat{\omega}$	0.1003	0.0062	0.0062	95.1
$A = 50000$	S_a	1096.8	29.8	(16.1) 29.9	(70.9) 95
$S = 6000$	$\hat{S}_A$	6006.6	198.2	(17.8) 199.3	(14.1) 95.2
	$\hat{S}_{Chao1}$	1558.3	68.7	60.3	0.0
	$\hat{S}_{ACE}$	1540.2	56.4	45.7	0.0
	$\hat{S}_{Jk1}$	1512.9	44.0	28.8	0.0
$\omega = 0.05$	$\hat{\omega}$	0.0501	0.003	0.003	95.2
$A = 50000$	S_a	1322.1	32.1	(16.5) 32.1	(68.8) 94.8
$S = 6000$	$\hat{S}_A$	6002.1	167.7	(17.4) 169.1	(16) 95
	$\hat{S}_{Chao1}$	1761.8	67.3	57.4	0.0
	$\hat{S}_{ACE}$	1719.0	53.4	40.6	0.0
	$\hat{S}_{Jk1}$	1736.2	45.2	28.8	0.0
$\omega = 0.01$	$\hat{\omega}$	0.01	6e-04	6e-04	95.1
$A = 50000$	S_a	1794.7	35.2	(23.2) 35.5	(80.3) 95.3
$S = 6000$	$\hat{S}_A$	5999.1	127.4	(17.9) 129.3	(21.6) 95.3
	$\hat{S}_{Chao1}$	2186.5	64.3	52.9	0.0
	$\hat{S}_{ACE}$	2128.2	50.8	35.2	0.0
	$\hat{S}_{Jk1}$	2179.6	46.0	27.7	0.0
$\omega = 0.005$	$\hat{\omega}$	0.005	3e-04	3e-04	94.5
$A = 50000$	S_a	1974	37.2	(24.6) 36.4	(79.9) 94.5
$S = 6000$	$\hat{S}_A$	5997.2	165.4	(17.9) 118.8	(21.7) 94.6
	$\hat{S}_{Chao1}$	2349.8	64.0	51.7	0.0
	$\hat{S}_{ACE}$	2290.7	51.3	34.1	0.0
	$\hat{S}_{Jk1}$	2344.4	47.4	27.2	0.0

Table S4: Simulation and estimation summarized from all the configurations when data were generated from truncated Poisson-lognormal distributions and total regional species richness S was fixed to 6000.

True value	Method	Average	Sample SE	Estimated SE	CP
Truncated Poisson-lognormal distribution: TPLN(0, 1.5)					
[redacted]	S_a	3613.3	37.7	(38.6) 47.6	(0) 0
$S = 6000$	$\hat{S}_A$	9705.1	152.6	(35) 146.1	(0) 0
	$\hat{S}_{Chao1}$	4736.9	88.9	84.1	0.0
	$\hat{S}_{ACE}$	4786.6	75.0	69.6	0.0
	$\hat{S}_{Jk1}$	4846.8	57.9	49.7	0.0
Truncated Poisson-lognormal distribution: TPLN(0, 2)					
[redacted]	S_a	3528.5	38.1	(42.4) 43.6	(0) 0
$S = 6000$	$\hat{S}_A$	7668.3	140.4	(31.1) 102.8	(0) 0
	$\hat{S}_{Chao1}$	4468.9	83.1	77.9	0.0
	$\hat{S}_{ACE}$	4443.2	67.6	59.5	0.0
	$\hat{S}_{Jk1}$	4539.0	55.2	44.9	0.0
Truncated Poisson-lognormal distribution: TPLN(0, 2.5)					
[redacted]	S_a	3458.4	37.9	(44.1) 40	(0) 0
$S = 6000$	$\hat{S}_A$	6449	137.5	(28.5) 78.5	(0.5) 2.2
	$\hat{S}_{Chao1}$	4266.4	81.8	73.0	0.0
	$\hat{S}_{ACE}$	4209.0	63.2	53.2	0.0
	$\hat{S}_{Jk1}$	4309.3	53.5	41.2	0.0
Truncated Poisson-lognormal distribution: TPLN(0, 3)					
[redacted]	S_a	3403.2	37.8	(44.6) 36.9	(0) 0
$S = 6000$	$\hat{S}_A$	5676.1	135.9	(26.7) 63.6	(1.1) 4.9
	$\hat{S}_{Chao1}$	4112.4	77.3	69.0	0.0
	$\hat{S}_{ACE}$	4043.7	60.5	48.9	0.0
	$\hat{S}_{Jk1}$	4137.7	52.0	38.3	0.0

Table S5: Simulation and estimation summarized from all the configurations when data were generated from truncated negative binomial distributions and total regional species richness S was fixed to 6000.

True value	Method	Average	Sample SE	Estimated SE	CP
Truncated negative binomial distribution: $\omega = 0.01$, $k = 1$					
[redacted]	S_a	5459.9	22.2	(31) 54.8	(0) 0
$S = 6000$	$\hat{S}_A$	12140.1	195.9	(31.8) 132.8	(0) 0
	$\hat{S}_{Chao1}$	5734.1	36.0	32.3	0.0
	$\hat{S}_{ACE}$	5660.0	26.5	19.4	70.8
	$\hat{S}_{Jk1}$	5956.3	32.3	31.5	70.8
Truncated negative binomial distribution: $\omega = 0.01$, $k = 0.5$					
[redacted]	S_a	4328.6	34.8	(29.4) 50.5	(0) 0
$S = 6000$	$\hat{S}_A$	10568.7	155.6	(30.8) 137.5	(0) 0
	$\hat{S}_{Chao1}$	4952.6	63.4	55.9	0.0
	$\hat{S}_{ACE}$	4854.1	48.0	37.4	0.0
	$\hat{S}_{Jk1}$	5177.5	48.9	41.2	0.0
Truncated negative binomial distribution:$\omega = 0.01$, $k = 0.1$					
[redacted]	S_a	2564.6	38.3	(24.1) 40.4	(0) 0
$S = 6000$	$\hat{S}_A$	7084.6	120.4	(26.3) 128.7	(0) 0
	$\hat{S}_{Chao1}$	3427.4	85.3	77.8	0.0
	$\hat{S}_{ACE}$	3374.9	68.8	57.5	0.0
	$\hat{S}_{Jk1}$	3424.8	56.1	41.5	0.0
Truncated negative binomial distribution: $\omega = 0.01$, $k = 0.01$					
[redacted]	S_a	2129.5	36.6	(22.3) 37.2	(0) 0
$S = 6000$	$\hat{S}_A$	6109.4	123.2	(24.7) 124.4	(21) 86.5
	$\hat{S}_{Chao1}$	3002.0	89.7	82.3	0.0
	$\hat{S}_{ACE}$	2967.8	72.4	62.3	0.0
	$\hat{S}_{Jk1}$	2927.3	55.0	39.9	0.0

Table S6: Simulation and estimation summarized from all the configurations when data were generated from truncated negative binomial distributions and total regional species richness S was fixed to 6000.

True value	Method	Average	Sample SE	Estimated SE	CP
Truncated negative binomial distribution: $\omega = 1$, $k = 1$					
[redacted]	S_a	3030.1	38.1	(28.5) 48.4	(78) 97
$S = 6000$	$\hat{S}_A$	13340	228.4	(40.7) 285.8	(0) 0
	$\hat{S}_{Chao1}$	4546.9	109.0	103.1	0.0
	$\hat{S}_{ACE}$	4667.4	102.4	98.3	0.0
	$\hat{S}_{Jk1}$	4544.9	63.5	55.0	0.0
Truncated negative binomial distribution: $\omega = 1$, $k = 0.5$					
[redacted]	S_a	1952.4	36.1	(22.4) 39.7	(0) 0
$S = 6000$	$\hat{S}_A$	10140.2	265.1	(36.8) 294.7	(0) 0
	$\hat{S}_{Chao1}$	3528.3	132.0	125.1	0.0
	$\hat{S}_{ACE}$	3649.2	131.5	124.1	0.0
	$\hat{S}_{Jk1}$	3130.6	62.1	48.5	0.0
Truncated negative binomial distribution: $\omega = 1$, $k = 0.1$					
[redacted]	S_a	1087	30	(15.9) 30.2	(0) 0
$S = 6000$	$\hat{S}_A$	6826.6	291.4	(31.3) 293.2	(0.2) 17.3
	$\hat{S}_{Chao1}$	2474.3	150.0	144.5	0.0
	$\hat{S}_{ACE}$	2568.8	152.2	147.4	0.0
	$\hat{S}_{Jk1}$	1844.3	53.3	38.9	0.0
Truncated negative binomial distribution: $\omega = 1$, $k = 0.01$					
[redacted]	S_a	918.8	27.8	(14.4) 27.9	(0) 0
$S = 6000$	$\hat{S}_A$	6092.4	293.3	(29.9) 292.7	(15.1) 94.7
	$\hat{S}_{Chao1}$	2236.8	155.8	149.5	0.0
	$\hat{S}_{ACE}$	2322.7	158.9	153.2	0.0
	$\hat{S}_{Jk1}$	1579.3	49.8	36.3	0.0
