# Supplementary material for: Rarefaction and extrapolation of species richness using an area‐based Fisher's logseries
Source: Ecol Evol. 2017 Oct 23;7(23):10066–78. doi: 10.1002/ece3.3509 (PMC5723611; doi:10.1002/ece3.3509)
Supplement: Supplementary file 2 [file ECE3-7-10066-s002.doc]

**Supporting Information**

**Rarefaction and extrapolation of species richness using an area-based Fisher’s logseries**

Youhua Chen^1,3^, Tsung-Jen Shen^2*^

1, Chengdu Institute of Biology, Chinese Academy of Sciences, Chengdu, 610000, China

2, Institute of Statistics & Department of Applied Mathematics, National Chung Hsing University, 250 Kuo Kuang Road, Taichung 40227, Taiwan

3, Department of Renewable Resources, University of Alberta, Edmonton, Alberta T6G 2H1, Canada

*Email for correspondence: [tjshen@nchu.edu.tw](mailto:tjshen@nchu.edu.tw)

Running title: area-based Fisher’s logseries

**Additional methods**

**A NBD degenerates at the zero point when *k* tends to zero**

To show this, we rewrite Eq. (1) of the main text in a tractable form as

$P(N=n)=\left\{\begin{matrix} \left(\frac{\omega}{\omega+1}\right)^{k},\text{ }n=0 \\ \left(\frac{k}{n}\right)\left(\frac{\omega}{\omega+1}\right)^{k}\left(\frac{1}{\omega+1}\right),\text{ }n=1 \\ \left(\frac{k}{n}\right)\left[\prod_{j=1}^{n-1} \frac{k+n-j}{n-j}\right]\left(\frac{\omega}{\omega+1}\right)^{k}\left(\frac{1}{\omega+1}\right)^{n},\text{ }n\geq2 \end{matrix}\right.$,

where the consecutive product of $P(N=n)$ for any $n\geq2$ approaches to one and $\left(1/(p+1)\right)^{k}\to1$ as $k\to0$. Consequently, in sum, $P(N=0)\to1$ while $P(N=n)\to0$ for any $n\geq1$ as $k\to0$. As a result, the probability mass function, Eq. (1) of the main text, degenerates at the zero point as $k\to0$.

**Simulation of the area-based Fisher’s logseries model**

Since the area-based logseries model is a standard probability function, whose random variates can be generated straightforwardly. Specifically, a random variate *u* from uniform distribution is generated first and compared to the cumulative probability function of the area-based logseries model. A species will have *n* individuals in the local sample *a* sampled from the regional area *A* if $F_{N}(n-1)<u\leq F_{N}(n)$.

**Rarefaction and extrapolation of species richness using Chao1, ACE and first-order Jackknife estimators and the calculation of variance**

The extrapolation of species richness by Chao1 estimator is

**,** (S1)

where $\hat{f}_{0}=f_{1}^{2}/(2f_{2})$ is the estimated number of unseen species in the whole population and $M_{a}+m$ is the number of individuals in the extrapolated sample. The variance of $\hat{S}_{Chao1}$ is calculated by the bootstrap method (Cha*o et a*l. 2014). For richness interpolation, Hurlbert (1971) suggested an unbiased estimator as

$\hat{S}_{Hurlbert}=S_{a}-\sum_{i\geq1} f_{i}\left(\begin{matrix} n-i \\ m \end{matrix}\right)/\left(\begin{matrix} n \\ m \end{matrix}\right),\text{ }m<M_{a}$ (S2)

The extrapolation of species richness by ACE estimator (Chao & Shen 2004) was

**,** (S3)

where

(S4)

and

$\hat{\gamma}^{2}=\max\left\{\frac{\sum_{i=1}^{10} f_{i}}{1-f_{1}/\sum_{i=1}^{10} f_{i}}\frac{\sum_{i=1}^{10} i(i-1)f_{i}}{\left(\sum_{i=1}^{10} if_{i}\right)^{2}}-1,\text{ }0\right\}$. (S5)

The variance of $\hat{S}_{ACE}$ was calculated by

, (S6)

where and **B** is the variance-covariance matrix of $\left(f_{1},\ldots,f_{10}\right)$. The (*i,j*)th element of **B** is explicitly expressed as follows:

. (S7)

For richness interpolation, Coleman (1981) suggested an area-based estimator as

$\hat{S}_{Coleman}=\sum_{i\geq1} f_{i}\left[1-\left(1-\frac{A^{*}}{a}\right)^{i}\right],\text{ }a\geq A^{*}$ (S8)

This is a classical form for species-area relationship. The variance estimator for this estimator has been suggested by Colwell et al. (2012) and was given by

. (S9)

Richness extrapolation by the first-order Jackknife estimator was given by

$\hat{S}_{Jk1}=S_{a}+f_{1}$ and whose variance estimator was , where and **B** has the same form as in Eq. S6 but with replacing $\hat{S}_{ACE}$ by $\hat{S}_{Jk1}$ in Eq. S7. Here, $\tau=\max\{k:f_{k},\text{ }k\geq1\}$. Again, richness interpolation is not available by using the Jackknife estimator.

**Original variance calculation method proposed by Fisher et al. (1943)**

Our proposed area-based model has an estimator of $V\hat{a}r\left(\hat{S}_{A^{*}}\right)$ as in Eq. 12 of the main text that is different from the one proposed by Fisher et al. (1943), whose variance formula was derived from fixing the number of individuals $M_{A^{*}}$ and given by

$V\hat{a}r\left(\hat{S}_{A^{*}}|M_{a}\right)=\hat{\alpha}\log\left(\frac{2M_{a}(A^{*}/a)+\hat{\alpha}}{M_{a}(A^{*}/a)+\hat{\alpha}}\right)-\frac{\hat{\alpha}^{2}M_{a}(A^{*}/a)}{\left(M_{a}(A^{*}/a)+\hat{\alpha}\right)^{2}}$. (S10)

Note that the original Fisher’s alpha can equivalently be estimated by $\hat{\alpha}=S_{A^{*}}/\log(1+A^{*}/\hat{\omega})$ using our area-based model. Remark that $V\hat{a}r\left(\hat{S}_{A^{*}}|M_{a}\right)\to\hat{\alpha}\log(2)$ as $A^{*}/a\to\infty$ (i.e., the extrapolated area size $A^{*}$ is large related to the local sample size *a*). This explains why Fisher’s original method failed to provide a reasonable estimated SE that is close to the sample SE for the regional species richness (Tables 2 and S1-S3).

**Theorem 1: The limiting distribution of $P(N_{a}=n|a,A,k,\omega)$ from Eq. (6) in the main text is in Eq. (7) when $k\to0$.**

**Proof:**

The probability function of TNBD is as follows:

$P(N_{a}=n|a,A,k,\omega)=\left\{\begin{matrix} \left(1-\left(\frac{\omega}{\omega+A}\right)^{k}\right)^{-1}\left[\left(\frac{\omega}{\omega+a}\right)^{k}-\left(\frac{\omega}{\omega+A}\right)^{k}\right],\text{ }n=0 \\ \left(1-\left(\frac{\omega}{\omega+A}\right)^{k}\right)^{-1}\frac{\Gamma(k+n)}{\Gamma(k)\Gamma(n+1)}\left(\frac{\omega}{\omega+a}\right)^{k}\left(\frac{a}{\omega+a}\right)^{n},\text{ }n=1,2... \end{matrix}\right.$.

For *n*=0, we can show

. (S11)

When *n* = 1, the limit probability is

. (S12)

For the other cases $n\geq2$, we derive the result from

, (S13)

where the consecutive product $\prod_{j=1}^{n-1} \frac{k+n-j}{n-j}\to1$ and $\left(\omega/(\omega+1)\right)^{k}\to1$ as $k\to0$.

Thus, we prove the desired result

.

**Theorem 2: geometric series model can be derived from TNBD in Eq. 3 of the main text when *k*=1.**

**Proof:**

When $k=1$, the TNBD can be transformed to the niche preemption model, which is a geometric series model as,

$\begin{matrix} P(N_{A}=n|A,k,\omega)=\frac{A}{\omega+A}\frac{\omega}{\omega+A}\left(1-\frac{\omega}{\omega+A}\right)^{n},\text{ }n=1\text{,2,}\ldots\\ \propto p(1-p)^{n-1} \end{matrix}$ (S14)

Where $p=\omega/(\omega+A)$.

**Theorem 3: TNBD in Eq. 3 of the main text predicts the highest number of singleton species when $k\to0$.**

**Proof:**

Define

$g(k)=P(N=1)=k\left(\frac{\omega}{\omega+A}\right)^{k}\left(\frac{A}{\omega+A}\right)\left[1-\left(\frac{\omega}{\omega+A}\right)^{k}\right]^{-1}$ , (S15)

then we have

$\begin{matrix} g'(k)=g(k)\left\{\frac{1}{k}+\log\left(\frac{\omega}{\omega+A}\right)+\frac{\left(\frac{\omega}{\omega+A}\right)^{k}\log\left(\frac{\omega}{\omega+A}\right)}{\left[1-\left(\frac{\omega}{\omega+A}\right)^{k}\right]}\right\} \\ \text{ }=g(k)\frac{1}{k}\left\{1+\frac{\log\left(\frac{\omega}{\omega+A}\right)^{k}}{\left[1-\left(\frac{\omega}{\omega+A}\right)^{k}\right]}\right\}<0 \end{matrix}$, (S16)

which is implied by the inequality $\log(1-x)+x<0$ for all $0<x<1$. Consequently, it is evident that $g(k)$ is a strictly decreasing function of $k$, where $k>0$. Thus, a lower $k$ will predict a higher number of singleton species.

Since the geometric series model is derived from TNBD when *k*=1 (see Eq. S14 from Theorem 2), it would predict a smaller number of singleton species in comparison to the proposed area-based Fisher’s logseries model in which $k\to0$. Theorem 3 applies to other rare species as well (e.g., doubletons).

**References**

Chao, A., Gotelli, N., Hsieh, T., Sander, E., Ma, K., RK, C. & Ellison, A. (2014). Rarefactionand extrapolation with Hill numbers: a framework for sampling and estimation in species diversity studies. *Ecological Monographs*, **84**, 45–67.

Chao, A. & Shen, T. (2004). Nonparametric prediction in species sampling. *Journal of Agricultural, Biological, and Environmental Statistics*, **9**, 253–269.

Coleman, B. (1981). Random placement and species-area relations. *Mathematical Biosciences*, **54**, 191–215.

Colwell, R., Chao, A., Gotelli, N., Lin, S., Mao, C., Chazdon, R. & Longino, J. (2012). Models and estimators linking individual-based and sample-based rarefaction, extrapolation and comparison of assemblages. *Journal of Plant Ecology*, **5**, 3–21.

Hurlbert, S. (1971). The nonconcept of species diversity: a critique and alternative parameters. *Ecology*, **52**, 577–586.

**Additional tables**

**Table S1.** Simulation and estimation summarized from all the configurations when total regional species richness *S* is fixed to 500.

**Table S2.** Simulation and estimation summarized from all the configurations when total regional species richness *S* is fixed to 2000.

**Table S3.** Simulation and estimation summarized from all the configurations when total regional species richness *S* is fixed to 6000.

**Table S4.** Simulation and estimation summarized from all the configurations when data were generated from truncated Poisson-lognormal distributions and total regional species richness *S* was fixed to 6000.

**Table S5.** Simulation and estimation summarized from all the configurations when data were generated from truncated negative binomial distributions and total regional species richness *S* was fixed to 6000.

**Table S6.** Simulation and estimation summarized from all the configurations when data were generated from truncated negative binomial distributions and total regional species richness *S* was fixed to 6000.
